# A machine learning approach for accurate and real-time DNA sequence identification

**DOI:** 10.1186/s12864-021-07841-6

**Published:** 2021-07-09

**Authors:** Yiren Wang, Mashari Alangari, Joshua Hihath, Arindam K. Das, M. P. Anantram

**Affiliations:** 1 Department of Electrical and Computer Engineering, University of Washington, 98195 Seattle, WA USA; 2 Electrical and Computer Engineering Department, University of California Davis, 95616 Davis, CA USA; 3 Department of Electrical Engineering, Eastern Washington University, 99004 Cheney, WA USA

**Keywords:** Single Molecule Break Junction, All-electrical detection, Conductance probability distribution, DNA sequence identification, Machine learning

## Abstract

**Background:**

The all-electronic Single Molecule Break Junction (SMBJ) method is an emerging alternative to traditional polymerase chain reaction (PCR) techniques for genetic sequencing and identification. Existing work indicates that the current spectra recorded from SMBJ experimentations contain unique signatures to identify known sequences from a dataset. However, the spectra are typically extremely noisy due to the stochastic and complex interactions between the substrate, sample, environment, and the measuring system, necessitating hundreds or thousands of experimentations to obtain reliable and accurate results.

**Results:**

This article presents a DNA sequence identification system based on the current spectra of ten short strand sequences, including a pair that differs by a single mismatch. By employing a gradient boosted tree classifier model trained on conductance histograms, we demonstrate that extremely high accuracy, ranging from approximately 96 % for molecules differing by a single mismatch to 99.5 % otherwise, is possible. Further, such accuracy metrics are achievable in near real-time with just twenty or thirty SMBJ measurements instead of hundreds or thousands. We also demonstrate that a tandem classifier architecture, where the first stage is a multiclass classifier and the second stage is a binary classifier, can be employed to boost the single mismatched pair’s identification accuracy to 99.5 %.

**Conclusions:**

A monolithic classifier, or more generally, a multistage classifier with model specific parameters that depend on experimental current spectra can be used to successfully identify DNA strands.

**Supplementary Information:**

The online version contains supplementary material available at 10.1186/s12864-021-07841-6.

## Background

DNA (Deoxyribonucleic acid) is one of the most essential and fundamental macromolecules for all forms of life. Therefore, the method to determine the arrangement of nitrogenous bases, DNA sequencing, has become an indispensable technique [1, 2]. Since the 1970 s, researchers have developed polymerase chain reaction (PCR) based methods to increase the throughput and accuracy and decrease both the time and cost of DNA sequencing. Current sequencing technologies are divided into short-read and long-read methods [3, 4]. Short-read methods have higher accuracy, lower cost, and smaller processing time [5]. However, they need the creation of many copies of the same DNA strand, a process known as amplification. Long-read methods can sequence a DNA molecule with more than 30,000 base pairs at once and are able to more accurately resolve complex regions of a DNA strand [6]. However, the accuracy of current long-read methods is still lower than short-read ones. The processing time and cost of the long-read methods are also disadvantaged compared to short-read methods [4]. Therefore, it has become critical to explore new methods to identify/sequence DNA strands. In this article, we demonstrate that “current data obtained from short-read sequences can be used for sequence identification with extremely high accuracies, without the need for amplification.”

Currently, no molecular detection and identification technique exists that can speedily, reliably, and without the need for amplification steps, detect or identify a low copy or a wide range of single biologically relevant molecules. A promising technique is an all-electronic method that would identify DNA or proteins based on the characteristic measurements of current [7]. This all-electronic DNA sequence identification system experiences nonlinear interactions between the substrate, sample, environment, and measuring system that are inherently stochastic [8, 9]. It has been extremely challenging for physics-based models to capture the differences in current between nominally different DNA strands. With the emergence of machine learning tools over the last decade, there is an increased interest in utilizing these methods to identify molecules from the current’s signature. Recently, surface-enhanced Raman spectroscopy [10], scanning tunneling spectroscopy (STS) [9, 11, 12], scanning tunneling microscopy break junction (STM-BJ) [13], and the measurement of ionic current blockade in nanopores [14] have been used to identify/sequence single-stranded DNA, RNA, and peptides. Identification of small molecules from a mixture was also recently demonstrated using machine learning classification methods [15–17].

In this paper, we demonstrate that double-stranded DNA molecules can be classified extremely accurately using machine learning methods operating on experimental quantum transport data. Typical classification accuracies for molecules which are structurally different exceed 99.9 %. Even in the case of DNA-RNA hybrids from E. coli with a single base pair mismatch, our methods are able to differentiate between them with an accuracy of over 96 %. The overall accuracy over ten datasets of different molecules is 98.85 %, with an average classification time of 27.49 µs (on a 2.2 GHz processor with 16 GB RAM) for each processed data input. However, we demonstrate that the accuracies of ‘problematic’ sequences such as S3 and S4 can be boosted to around 99.5 % if a two-stage classifier (a multiclass model cascaded with a binary model) is employed instead of a single-stage classifier. Our analysis and simulation results demonstrate the potential of combining current spectra and ML methods as a diagnostic tool for real-time detection and classification of genetic sequences.

## Results and discussion

### Overview of classification approach

**Fig. 1:**
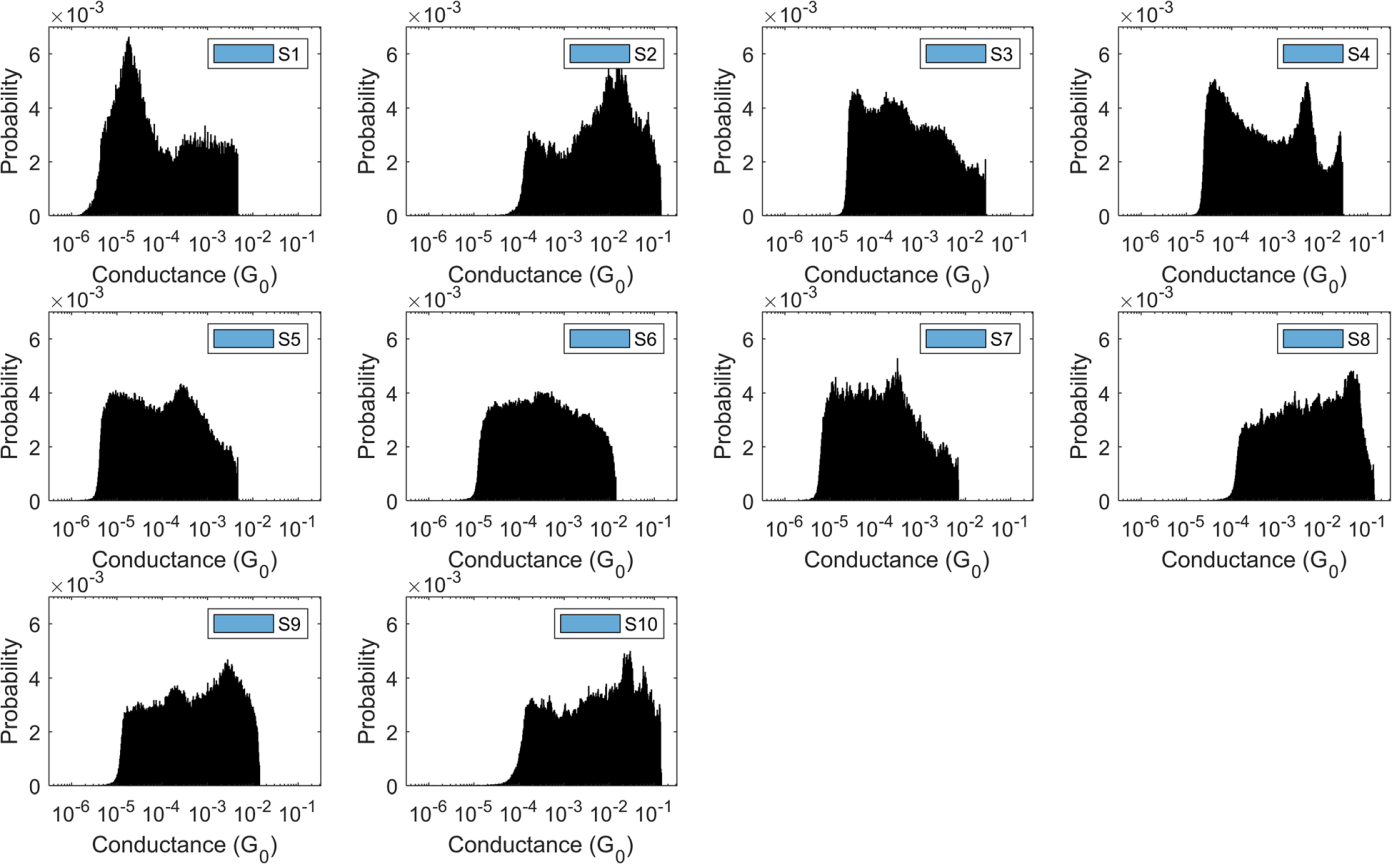
Large sample histograms of all data classes. The *R*^2^ test is: ‘accept current trace if *R*^2^ ≤ *β* = 0.95’. After the current traces are *R*^2^ filtered, one histogram is constructed per class, using all available current traces, which are converted to conductance traces after low pass filtration (see Figures S2 and S3 for details)

Raw current traces from SMBJ experiments [18] (Supplementary Figure S1) are first converted to conductance traces through a series of pre-processing steps, details of which are available in Supplementary Information Secs. 1 and 2. Histograms are then derived from the conductance traces. Figure 1 shows the empirical *large sample* conductance histograms for our ten datasets (see Table 1 for details) using an *R*^2^ test threshold of *β* = 0.95. We refer to these histograms as large sample histograms since these are based on thousands of conductance traces for each dataset (see Supplementary Table S11 for number of traces). From a practical perspective, however, strand level identification needs to be made from reasonably accurate histograms constructed from the fewest number of conductance traces possible. This is necessary to avoid imposing a costly experimental burden, which can potentially detract from adoption of current-based methods for applications which demand almost real-time genetic sequence determination.

**Table 1:** Details of the ten datasets used in this paper

Label	Sequence	Bias	Note	Solvent / Buffer
S1	Octanedithiol	0.30 V	Not a DNA/RNA	mesitylene
S2	5’-CCC GGG CCC GGG-3’ 3’-GGG CCC GGG CCC-5’	0.01 V		100mMP + 100uL + 30uL
S3	5’-CGA CCC CTC UUG AAC-3’ 3’-GCT GGG GAG AAC TTG-5’	0.05 V	E. coli O157:H7	10uL + 75 μm + 600MC_50BC_20RR
S4	5’-CGA CCC CTC UUG AGC-3’ 3’-GCT GGG GAG AAC TTG-5’	0.05 V	E. coli O175:H28 One mismatch from S3	30ul + 7.5 μm + Rg
S5	5’-CGA CCC CCC UUG AAC-3’ 3’-GCT GGG GAG AAC TTG-5’	0.30 V	E. coli ED1a One mismatch from S3	75 μm + 10uL
S6	5’-CCC GGG CCC GGG-3’ 3’-GGG CCC GGG CCC-5’	0.10 V	Same as S2	100mMP + 100uL + 30uL
S7	5’-CCC GGG CCC GGG-3’ 3’-GGG CCC GGG CCC-5’	0.20 V	Same as S2	100mMP + 100uL + 30uL
S8	5’-CCC GGG CCC GGG-3’ 3’-GGG CCC GGG CCC-5’	0.01 V	Same as S2	100mMP + 100uL + 20uL
S9	5’-CCC GGG CCC GGG-3’ 3’-GGG CCC GGG CCC-5’	0.10 V	Same as S2	100mMP + 100uL + 50uL
S10	5’-GGG TTT GGG-3’	0.01 V	G-quadruplex secondary structures	100mMP + 100uL + 30uL

**Fig. 2:**
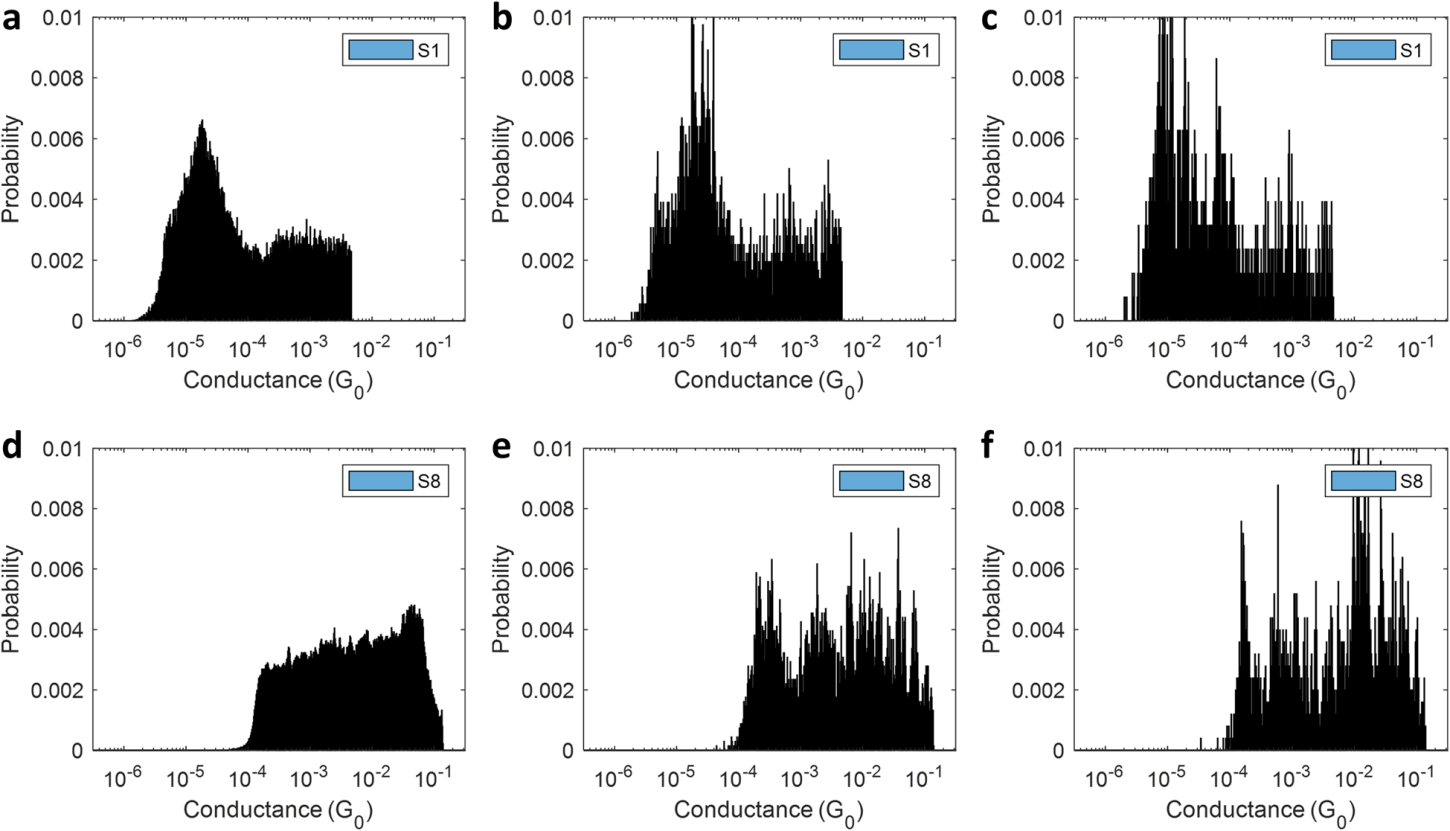
Comparison of large sample, baseline, and small sample histograms.(**a**)**, **(**d**) Large sample, (**b**), (**e**) baseline (*H* = 30) and **(c), (f)** small sample (*H* = 10) conductance probability histograms for datasets S1 **(a-c)** and S8 **(d-f)**

Ideally, we would like to conduct a successful (with molecular binding) experiment once and be able to predict the strand from the resulting conductance values. However, given the substantial noise and uncertainty inherently associated with the current state of SMBJ experimentation, a compromise is necessary. We train and evaluate our classifiers based on *H*-sample conductance histograms - histograms constructed from randomly sampled *H* ‘valid’ conductance traces (whether a trace is deemed valid or not is determined by the *R*^2^ test threshold *β*) - and study the impact of the parameter *H* on classifier accuracy. Henceforth, we will refer to histograms constructed from reasonably small values of *H*, *e.g. H* ≤ 20, as *small sample* histograms. Additionally, we define any histogram based on *H* = 30 as a *baseline* histogram. Figure 2 shows a representative large sample, baseline, and small sample (*H* = 10) histograms for datasets S1 and S8. We observe that while the conductance distribution of S8 changes significantly at *H* = 30 (note the multiple peaks in the conductance range $\left[{10}^{-4}, {10}^{-2}\right] {G}_{0}$), the distribution of the baseline histogram for S1 appears to be relatively consistent with its large sample counterpart. The significant change in the conductance histogram for S8 at *H* = 30 probably alludes to unique contact configurations between DNA and electrodes. Nevertheless, expecting consistently perfect experimental conditions is a luxury in practice and classifier models need to be relatively robust to such uncertainties.

Starting with ten datasets, we trained classifier models using two different target class labeling schemes. In the first scheme (TLS-1), unique DNA strands were assigned different class labels, irrespective of the voltage bias used for current measurement during SMBJ experiments, resulting in six target classes. In the second scheme (TLS-2), the datasets are assigned unique class labels based on the (strand, voltage bias) tuple, resulting in eight classes. Additional details and justification for using the different labeling schemes are available in Supplementary Information Sec. 2.1. For both labeling schemes, we randomly partitioned our conductance traces, 70 % for training and 30 % for testing. To reduce any potential bias due to the initial split, each simulation was repeated 100 times with a random train/test split. For each simulation, *H* traces were sampled randomly from each class to generate a conductance histogram. This process was repeated for a total of *N*_*hist*_ = 700 times per class, where *N*_*hist*_ is the number of histograms. The total number of training samples is, therefore, 4200 for TLS-1 (six classes) and 5600 for TLS-2 (eight classes). A similar procedure was adopted for evaluating classifier performance, except we chose *N*_*hist*_ = 300 per class. The total number of testing samples is therefore 1800 for TLS-1 and 2400 for TLS- 2. All accuracy metrics reported in this paper are averaged over 100 random test splits.

We experimented with three different classification paradigms, (i) multilayer perceptron artificial neural network (ANN), (ii) support vector machine (SVM) with a radial basis function kernel, and (iii) extreme gradient boosting (XGboost). Since the performances of ANN and SVM methods were generally at par with or slightly worse than XGboost, we will concentrate on the performance of the boosted method in this article. Broadly speaking, XGboost [19] is a fast and scalable implementation of a gradient boosted decision tree framework [20]. Gradient boosting is an ensemble learning method wherein weak base learners (usually decision trees) are added sequentially, one at each iteration, to minimize a suitably defined loss function evaluated on the previous learner. Within XGboost, the loss function is cross entropy for multiclass classification problems. For details on gradient boosting in general, we refer the reader to Hastie’s article [21]. We used the Python implementation of the XGboost package [22]. Two critical hyperparameters within an XGboost framework are the number of trees/estimators, *N*_*est*_, and the depth of each tree/estimator, *D*_*est*_. Based on extensive hyperparameter optimization, we chose *N*_*est*_ = 200 and *D*_*est*_ = 2. Additional details can be found in the Methods section.

Let *N*_*bins*_ denote the number of bins for a conductance probability histogram. We define a *baseline classifier* as one which is characterized by the following parameters: (i) *N*_*bins*_ = 600 (i.e., each training sample is a 600 dimensional probability vector), (ii) *R*^2^ ≤ *β* = 0.95 in the pre-processing sequence, and (iii) *H* = 30. On a finer note, we add that the first and last bins of the histograms were removed to avoid any spikes induced by current clipping in the first pre-processing step (see Supplementary Figures S2 and S3). Therefore, the actual number of histogram bins used is 598, or in general, *N*_*bins*_ – 2, whatever be the value of *N*_*bins*_ reported in this paper.

In the subsequent sections, we first discuss the performances of the baseline classifiers for both target class labeling schemes, followed by an analysis of their sensitivities to the choice of *N*_*bins*_, *β*, and *H*. Irrespective of the choice of *N*_*bins*_, *β*, and *H*, we have consistently used *N*_*hist*_ = 700 per class for training and *N*_*hist*_ = 300 per class for testing. All classifier accuracy results reported are averaged over 100 evaluations. Finally, we refer the reader to Supplementary Information Sec. 3 for *t*-SNE and multidimensional scaling maps of our data. These low dimensional visualizations provide broad insights into the structure of the data and are useful in deriving insights into classifier performance.

### Performance of baseline classifiers

**Fig. 3:**
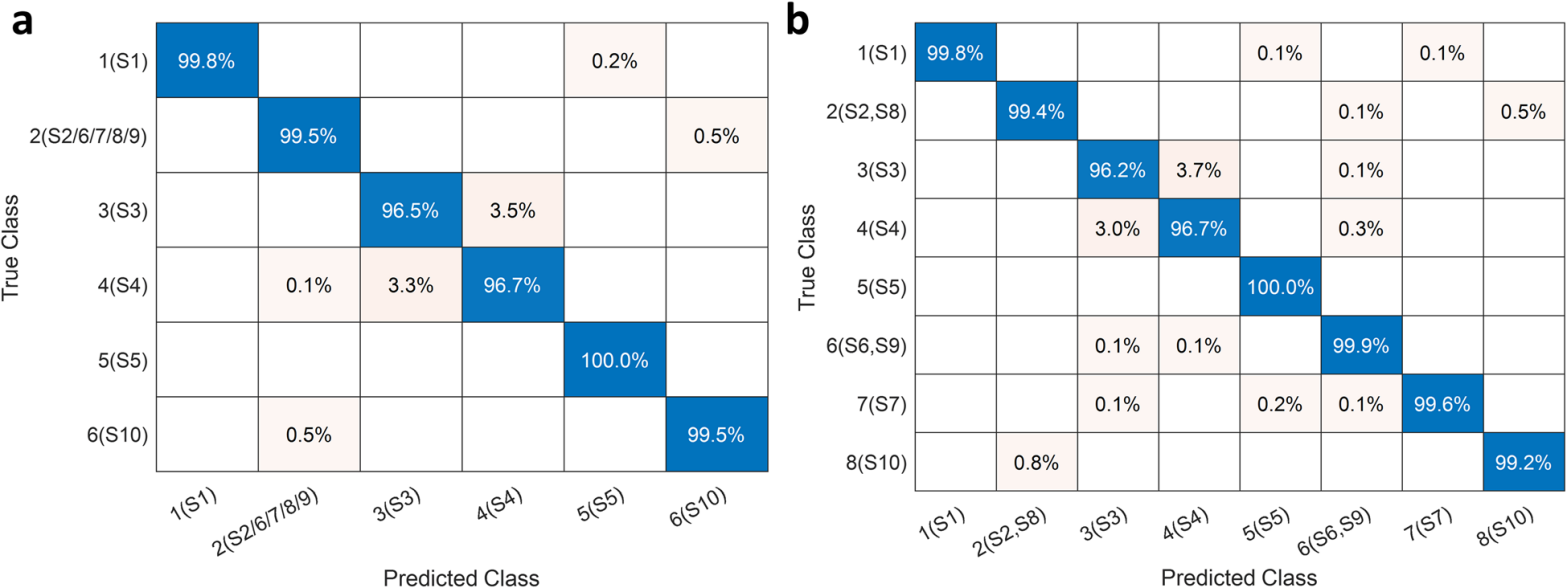
Confusion matrices for baseline classifiers. (**a**) Confusion matrices corresponding to target labeling scheme TLS-1 with 6 classes. (**b**) Confusion matrices corresponding to target labeling scheme TLS-2 with 8 classes

Figure 3 shows the confusion matrices of the baseline classifiers for both target class labeling schemes. We recall that datasets S2, S6, S7, S8, and S9 pertain to the same DNA strand, though with three different bias voltages. First, we observe that classifier accuracies for these five datasets are not affected by choice of the labeling scheme. Highly accurate strand level identification is possible whether the data is labeled based purely on strand type or a finer (strand type, bias voltage) tuple. Any ML framework should be able to utilize data from instrumentation that uses less sensitive current amplifiers. Lower sensitivity current amplifiers require a larger bias to record current. As the bias increases, molecular conformation may change in manners that are not yet fully understood or characterized. Consequently, the ability to detect a strand independent of the bias applied is invaluable. Second, we observe that classes S3 and S4 can be differentiated with an accuracy exceeding 96.5 %, despite the fact that S4 is just a single mismatch mutant of S3. Although TLS-2 seems to enjoy a slight edge in performance as far as S3 and S4 are concerned, the difference is not appreciable to claim a definite advantage for any particular labeling scheme. Third, we note that class S5 poses no issues for the baseline classifiers and is always identified perfectly. Fourth, whenever (S2, S8) is misclassified, it is predicted to be S10, and vice versa. This is consistent with the low dimensional visualizations shown in Supplementary Figures S4 and S5. Finally, we observe that, barring S3 and S4, the XGboost baseline classifiers work phenomenally well with individual class accuracies exceeding 99.5 % for *H* = 30. For S3 and S4, the accuracies are within 96.2–96.7 %. Our investigations indicate that instead of a single classifier, a tandem classifier architecture, consisting of a primary classifier involving all classes and a secondary binary classifier operating only on S3 and S4 (see Supplementary Table S2), can significantly boost the prediction accuracies of classes S3 and S4 to approximately 99.5 %. Additional details are available in Supplementary Information Sec. 4 (see in particular, Supplementary Figures S6 and S7).

### Performance analysis of baseline classifiers with respect to the ***R***^2^ test threshold parameter, ***β***

**Fig. 4:**
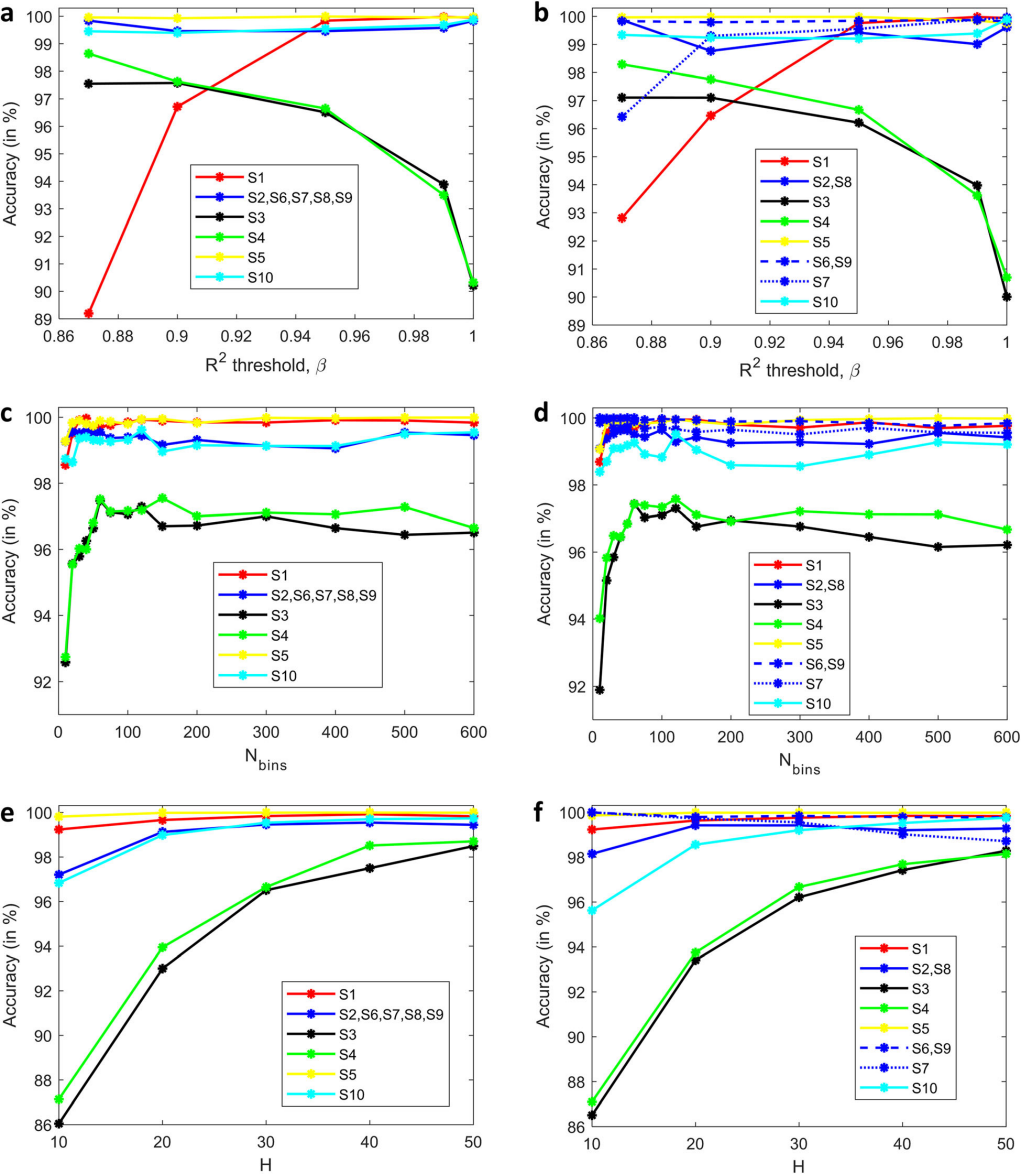
Performance analysis of baseline classifiers with respect to (w.r.t) β, Nbins, and H. (**a**), (**b**) Accuracy of baseline classifiers w.r.t the R2 test threshold parameter, β, corresponding to target labeling scheme TLS-1 with 6 classes (**a**) and TLS-2 with 8 classes (**b**). We chose the same color but different line types to distinguish between datasets [S2,S8], [S6,S9], and [S7], which are of the same strand but use different bias voltages. (**c**), (**d**) Accuracy of baseline classifiers w.r.t the number of histogram bins, Nbins, corresponding to target labeling scheme TLS-1 with 6 classes (c) and TLS-2 with 8 classes (**d**). Similar color scheme and line types as in (**a**, **b**) have been used to distinguish between datasets [S2,S8], [S6,S9], and [S7]. (**e**), (**f**) Accuracy of baseline classifiers w.r.t the number of traces used to compute a conductance histogram, H, corresponding to target labeling scheme TLS-1 with 6 classes (**e**) and TLS-2 with 8 classes (bs). We chose the same color but different line types to distinguish between datasets [S2,S8], [S6,S9], and S7, which are of the same strand but use different bias voltages.

Figure 4(a) and (b) show the accuracy of the baseline classifiers with respect to (w.r.t) the *R*^2^ test threshold parameter, *β*, corresponding to the two different target labeling schemes, maintaining *N*_*bins*_ = 600 and *H* = 30 (for detailed confusion matrices, see Supplementary Figure S8). Intuitively, we should expect a higher number of invalid (no molecule) or somewhat invalid (traces which are substantially similar to an exponential decay) traces to be rejected for lower values of *β*. Provided an adequate number of traces are retained after this step, this should yield a clean pool of valid traces to sample from, which should translate to improved or similar classifier accuracies. However, we observe that the accuracy for S1 drops significantly for lower values *β* with both labeling schemes, as does the accuracy for S7 for TLS-2. This can be explained by the fact that only 103 and 207 current traces remain for S1 and S7 respectively, when *β* = 0.87. Even though *N*_*hist*_ = 700 for all classes during training, the shallow pool of traces to sample from means that a substantial number of the training histograms for these two classes are reasonably similar and do not contribute to classifier learning. On the other hand, datasets S3 and S4 show an almost 8 % improvement in accuracy as *β* is lowered from 1.0 to 0.87. This result can be further understood by observing the *t*-SNE visualization (see Supplementary Figure S4), which shows a mostly clean separation between the S3 and S4 clouds at *β* = 0.87. At the lower end of *β*, 532 and 874 current traces remain for S3 and S4 respectively, enough for meaningful repeated sampling. We postulate that, for reasonably high values of *β*, small/medium sample training histograms of these two classes are somewhat contaminated by invalid or substantially invalid traces, which masks out the true differences we expect between the two classes. A lower value of *β* which aggressively filters out raw traces, decreases the odds of histogram contamination, and allows the classifier to discover subtle differences, leading to a substantial enhancement in accuracy. Therefore, we conclude that a ‘*one β fits all*’ policy need not be the most prudent choice for all classes of data.

### Performance analysis of baseline classifiers with respect to the number of histogram bins, ***N***_***bins***_

Figure 4(c) and 4(d) show the accuracy of the baseline classifiers w.r.t the number of histogram bins, *N*_*bins*_, corresponding to the two different target labeling schemes, maintaining *β* = 0.95 and *H* = 30 (for detailed confusion matrices, see Supplementary Figure S9). Typically, histograms with small bin-widths are preferred because they closely resemble the underlying probability distribution. This is affirmed in Fig. 4(c) and 4(d), where we observe that the accuracies are generally highest when the number of bins is large (600), except for classes 3 and 4. For these two classes, we observe that a coarse binning strategy with just 60 bins has better accuracy for both labeling schemes, which is evidence that ‘*finer is not always better*’ when it comes to choosing the granularity of the conductance histograms. While we do not have a definite explanation for why a fewer number of bins is better for S3 and S4, we conjecture that a fine binning strategy, especially when coupled with smallish values of *H*, generates ‘pseudo-features’ which are detrimental to learning by overfitting due to *curse of dimensionality*. We conjecture that a coarse binning strategy with its inherent noise averaging properties appears to avoid this issue and maybe a better choice for distinguishing between molecules which differ in one or few mismatches.

It is also interesting to note that *N*_*bins*_ = 200 appears to be a reasonable choice for all classes for both labeling schemes, except S3, S4, and S10. While we have addressed the issues with (S3, S4) in the preceding paragraph, we hypothesize that the increased accuracy of S10 as *N*_*bins*_ increases is related to the low-dimensional overlap of S2, S8, and S10, as illustrated in Supplementary Figures S4 and S5. Stated differently, these three classes benefit from a sufficiently high number of dimensions (bins), which allows the classifier to narrow in on the fine differences between (S2, S8) and S10. In general, coarse binning has the effect of smoothing out these subtle differences, resulting in a drop of class accuracy.

### Performance analysis of baseline classifiers with respect to the number of traces used to compute a conductance histogram, ***H***

Figure 4(e) and 4(f) show the accuracy of the baseline classifiers w.r.t the number of traces used to compute a conductance histogram, *H*, corresponding to the two different target labeling schemes, maintaining *N*_*bins*_ = 600 and *β* = 0.95 (for detailed confusion matrices, see Supplementary Figure S10). In this case, we expect ‘*more is better*’ from a performance perspective when it comes to choosing a proper value for *H*, since histograms constructed from a sufficiently large number of traces should more closely approximate the underlying conductance distribution. This is corroborated in Fig. 4(e) and 4(f), with the only exception being S7 for TLS-2. For this class, the accuracy shows a consistently decreasing trend, dropping from almost 100–98.9 % as *H* is increased from 10 to 50. From our simulations, we find that the misclassification probabilities for S7 are 0.009 and 0.011 for *H* = 40 and 50 respectively, and in both cases, the probability that S7 is predicted to be S5 is 0.007, based on results averaged over 100 runs. Therefore, whenever S7 is misclassified, about 70 % of the time, it is incorrectly predicted to be S5. At first glance, the reason for this exception might appear to be related to data scarcity since dataset S7 has 538 valid conductance traces to sample from at *β* = 0.95. With larger values of *H* such as 40 or 50, it is plausible that the corpus of training histograms for S7 is not diverse enough for the ensemble classifiers to properly learn to distinguish S7 from other classes in the database. However, if we observe the accuracy of S1, it does not exhibit a similar performance degradation as S7, despite the fact that only 528 valid traces remain at *β* = 0.95. An explanation for this contradictory behavior can be derived from a visual examination of the large sample conductance histograms shown in Fig. 1. To the naked eye, the large sample histograms for S5 and S7 appear somewhat similar, while S1 stands out from all other datasets due to its pronounced peak at a conductance value of approximately${ 10}^{-6} {G}_{0}$. For smaller values of *H*, we believe that the inherent small sample variability induces small differences (which can be viewed as additive noise) between S5 and S7 histograms, which aids with classifier generalization. Stated differently, we suspect that the XGboost classifier may be overfitting on S7 at larger values of *H*, which could be compounded by the data scarcity issue. On the other hand, we hypothesize that the acuity of the signature dominant peak at ${10}^{-5} {G}_{0}$ for S1 is relatively immune to the choice of *H*, which ensures that its classification accuracy remains consistently high across *H*.

While a higher value of *H* may be generally better from a performance perspective, it imposes an undue burden in practice since an SMBJ experiment needs to be conducted multiple times to make a highly accurate visual prediction. Specifically, if the probability of any experiment being valid (with molecular binding) is *p*, which of course depends on the *R*^2^ test threshold parameter *β*, the average number of times an experiment needs to be conducted is *H*/*p*.

## Conclusions

Identification of genetic material from signatures in current traces has been pursued for over a decade. The conductance histograms from experimentally measured currents are intrinsically noisy, with values ranging over three orders of magnitude. Further, physics-based theory and modeling approaches have so far been ineffective in capturing the noisy nature of experimental data with enough accuracy to reveal sequence information. This is because the number of atoms involved is extremely large, the environment fluctuates, and there are a large number of DNA-contact configurations, which makes it impossible to model the system in a realistic manner. As a result, identifying genetic material from current spectra has remained a challenge. In this work, we have demonstrated that a ML based approach using experimentally obtained current spectra is extremely successful in identifying DNA strands. Conductance traces were sampled randomly (*H* at a time) to construct the conductance histograms and an XGboost classifier was trained on the histograms. The computational time for strand identification from raw experimental data using a trained model is as small as 28 µs, which makes our approach suitable for real-time implementation. For molecules which are sufficiently different structurally, we obtain an accuracy of 99.8 % with *β* ≥ 0.95 and *H* as small as 20–30. For DNA-RNA hybrids from E. coli with just a single base pair mismatch, our methods can achieve similar accuracy, but with a more stringent set of parameters, *β* = 0.90 and *H* = 50. In general, we observe that a monolithic classifier model trained on multiple data classes may not provide comparably high accuracy for genetic samples which differ by a single mismatch, especially if *H* is kept reasonably low in the interest of rapid detection and classification in practice. Our investigations reveal that a tandem classifier approach, where the first stage is a multiclass classifier and the second stage is a binary classifier operating exclusively on molecules with single base pair mismatches, can be an attractive architectural proposition for boosting the accuracies of such samples to around 99.5 % with a reasonable *H* = 30. Overall, our approach shows tremendous potential for accurate, fast, cheap, and amplification free DNA strand identification. Extremely short computational times, along with demonstrated high accuracies, makes single-molecule sensing using the current spectra possible in real-time and establishes it as a feasible candidate for diverse time-critical sequence identification applications, including clinical diagnostics.

## Methods

### Data acquisition

The SMBJ experiments are conducted at room temperature using a Molecular Imaging Pico-STM. The STM probe is connected to a Digital Instruments Nanoscope IIIa controller which records the current traces. For a more detailed experiment setup, please refer to [18]. Subsequently, the current traces are converted to conductance traces, details of which are available in Supplementary Information Secs. 1 and 2.

For each experiment, a small volume of RNA:DNA hybrid molecule is first injected between two gold electrodes (the tip and substrate), meeting a desired concentration level. A bias voltage within the range of 50–300 mV is applied between the two gold electrodes. The STM probe, which is controlled by the LabView program, is then moved towards the substrate at a rate of approximately 80 nm/s until the current saturates the preamplifier (~ 100 nA). The tip is then retracted at the same rate until the current reaches the lower limit of the preamplifier (~ 10 pA). The whole process is repeated until the tip reaches the edge of the substrate. Among the thousands of collected traces, a significant number shows a predominantly exponentially decaying form, which implies that no molecules were captured between the tip and the substrate (invalid experiment). A small number of current traces shows steps and flat regions (in general, substantial deviations from an exponentially decaying characteristic), which is indicative of a successful molecular binding between the tip and the substrate.

### Data pre-processing

A series of pre-processing steps were used to convert the experimentally obtained SMBJ current traces to conductance histograms. Since the recorded current values are the outputs of a preamplifier, which has a noise floor of 10 *p*A and an upper limit of 100 *n*A, all current traces were first limited to the threshold range [10 *p*A − 100 *n*A].

Due to uncertainties in the data acquisition process, sometimes an experiment is conducted even when no molecule is attached to the electrode. Such experiments are deemed to be invalid. Invalid current traces exhibit a predominantly exponential decay over time. Since our datasets had a mix of valid and invalid traces for each molecule, in the second pre-processing step, we fit an exponential model to the current traces and adopted an *R*^2^ test with a suitable threshold to accept/reject a trace.

In the third pre-processing step, all accepted current traces were low pass filtered. Traces for data classes S1 to S9 were filtered with a cutoff frequency of 9 kHz, while those for data class S10 were filtered with a cutoff frequency of 3 kHz. In both cases, the cutoff frequencies correspond to 60 % of the folding frequencies (half the sampling rate). Since the sampling rate during the data acquisition phase for S10 was 10 kHz., compared to 30 kHz. for S1−S9, all S10 current traces were linearly interpolated by a factor of 3 after low pass filtration.

Finally, the processed current traces were converted to conductance traces, which were then sampled randomly to generate the conductance histograms normalized to probabilities. Additional details on the pre-processing phase are available in Supplementary Information Sec. 2.

### XGboost

We used the Python implementation of the XGboost package available at^19^. Two critical hyperparameters within XGboost are the number of trees/estimators, *N*_*est*_, and the depth of each tree/estimator, *D*_*est*_. Optimal values of these parameters were determined based on an exhaustive grid search on values indicated below:

$$ < mathdollar>{\displaystyle \begin{array}{c}{D}_{est}=1,2,3,4,5,6,7,8,9,10,11,12,13,14,16,18,20\\ {}{N}_{est}=10,50,100,150,200,250,300,350,400,500,600,700\end{array}} $$

Based on validation accuracies, we determined that reasonable values are (*N*_*est*_ = 200, *D*_*est*_ = 2) or (*N*_*est*_ = 150, *D*_*est*_ = 3). For all simulations, we chose the first parameter combination to reduce the computational time. All other parameters were left at their default values. In particular, the default value of the learning rate or shrinkage parameter is 0.3.

## Supplementary Information

**Additional file 1:** **Table S1**. Data availability after *R*^2^ test. **Table S2**. Class accuracies in percentages for a secondary binary SVM classifier operating on S3 and S4. **Figure S1**. Raw experimental input data. **Figure S2**. Sequence of pre-processing steps for converting raw current traces to conductance traces. **Figure S3**. Representative large sample conductance histograms for data class S1 using threshold parameter *β* = 1. **Figure S4**. *t*-SNE visualization of our datasets. **Figure S5**. Classical multidimensional scaling (MDS) visualizations. **Figure S6**. Clustering of S3 and S4 data. **Figure S7**. Binary SVM classifier on S3 and S4. **Figure S8**. Performance analysis of baseline classifiers with respect to the *R*^2^ test threshold parameter, *β. ***Figure S9**. Performance analysis of baseline classifiers with respect to number of histogram bins, *N*_*bins*_. **Figure S10**. Performance analysis of baseline classifiers with respect to the number of traces used to compute a conductance histogram, *H.*

## Data Availability

The datasets used and analysed during the current study are available from the corresponding author on reasonable request.

## Abbreviations

ANN: Artificial neural network
DNA: Deoxyribonucleic acid
PCR: Polymerase chain reaction
SMBJ: Single molecule break junction
STM-BJ: Scanning tunneling microscopy break junction
STS: Scanning tunneling spectroscopy
SVM: Support vector machine
XGboost: Extreme gradient boosting
