# Supplementary material for: A machine learning approach for accurate and real-time DNA sequence identification
Source: BMC Genomics. 2021 Jul 9;22:525. doi: 10.1186/s12864-021-07841-6 (PMC8268518; doi:10.1186/s12864-021-07841-6)
Supplement: Supplementary file 1 — Additional file 1: Table S1. Data availability after R2 test. Table S2. Class accuracies in percentages for a secondary binary SVM classifier operating on S3 and S4. Figure S1. Raw experimental input data. Figure S2. Sequence of pre-processing steps for converting raw current traces to conductance traces. Figure S3. Representative large sample conductance histograms for data class S1 using threshold parameter β = 1. Figure S4. t-SNE visualization of our datasets. Figure S5. Classical multidimensional scaling (MDS) visualizations. Figure S6. Clustering of S3 and S4 data. Figure S7. Binary SVM classifier on S3 and S4. Figure S8. Performance analysis of baseline classifiers with respect to the R2 test threshold parameter, β. Figure S9. Performance analysis of baseline classifiers with respect to number of histogram bins, Nbins. Figure S10. Performance analysis of baseline classifiers with respect to the number of traces used to compute a conductance histogram, H. [file 12864_2021_7841_MOESM1_ESM.docx]

**Supplementary Information**

**A Machine Learning Approach for Accurate and Real-Time DNA Sequence Identification**

Yiren Wang^1*^, Mashari Alangari^2^, Joshua Hihath^2^, Arindam K. Das^3^, M. P. Anantram^1*^

^1^Department of Electrical and Computer Engineering, University of Washington, Seattle, WA 98195, USA. ^2^Electrical and Computer Engineering Department, University of California Davis, Davis, CA 95616, USA. ^3^Department of Electrical Engineering, Eastern Washington University, Cheney, WA 99004, USA.

*Corresponding author: Yiren Wang, M. P. Anantram

**Email:** ethanwyr@uw.edu, anantmp@uw.edu

1. **Data acquisition.**

The Single Molecule Break Junction (SMBJ) approach consists of a molecule binding to a conducting substrate on one end and a scanning microscope tip on the other end. Current flow between the tip and substrate is measured as a function of time. Figure S1(a) shows an idealized schematic of the SMBJ experimental setup. Representative current traces without and with DNA molecular binding between the gold electrode and the substrate are shown in Figures S1(b) and S1(c) respectively. In the absence of any binding, the current trace exhibits a predominantly exponential decay as the electrode is moved away from the substrate. Deviations from this behavior as shown by increases/dips in Figure S1(c) are generally indicative of a successful molecular binding and a ‘valid’ experiment. Our hypothesis is that unique signatures of the DNA molecule exist in the conductance (ratio of current to applied voltage) traces, specifically, conductance histograms (see Figures 1 and 2 in the main article), and automated classification of DNA strands should be possible using statistical and/or machine learning (ML) based approaches.

We use ten datasets of experimentally obtained current traces to validate our ML approach. Each dataset contains a mix of valid (with molecular binding) and invalid (no molecular binding) traces. In the next section, we explain how the invalid traces were automatically pre-filtered prior to classifier training. Table 1 in the main article provides the details of the ten datasets. Some salient features of the datasets are as follows. First, although S2, S6, S7, S8, and S9 are of the same strand, tests were conducted using three different bias voltages. Second, S4 and S5 are mismatched versions of S3, which corresponds to mRNA from E. coli: O157:H7 with its fully matched DNA duplex and is known to produce both Shiga toxins (Stx) 1 and 2. S4 and S5 have the same DNA complement as S3. The mRNA from S4 corresponds to E. coli O175:H28. It has a single mismatch at base 14 of S3 (A is substituted by G) and is known to lead to Stx 2. The mRNA from S5 which corresponds to E. coli E1a, has a single mismatch at base 8 of S3 (C is substituted by T) and is nontoxic as it does not produce either Stx 1 or 2.

1. **Data pre-processing.**

Some pre-processing steps were carried out to convert the experimentally obtained ‘raw’ current traces to suitable conductance traces which are used to derive the conductance histograms for training our ML models. Since the recorded current values are the outputs of a preamplifier which has a noise floor of 10 *p*A and an upper limit of 100 *n*A, we first clipped all current traces in our database to the range [10 *p*A − 100 *n*A].

Next, we fit an exponential regression model, $y=a\cdot e^{-b\cdot x}, b>0,$ to each clipped current trace and used the *R*^2^ statistic from the fit to accept/reject the trace. Specifically, if the computed *R*^2^ statistic is greater than some chosen threshold, *β*, we reject the trace as `invalid’ (experiment conducted with no molecular binding); otherwise, the trace is accepted. Even though the standard error of regression is a better choice for nonlinear regression models, we determined through extensive experimentation that the *R*^2^ statistic works well for our purposes. Starting with about 1400−7000 traces for each of our 10 data classes, we found that approximately 50−90% of the data were rejected at this step for *β* = 0.95, 70-96% of the current traces were rejected for *β* = 0.90, and 78-98% of the current traces were rejected for *β* = 0.87 (see Table S1 below). Given the scarcity of experimental data, a prudent choice of *β* is necessary to retain as much data as possible, while rejecting the most obviously invalid current traces.

**Table S1. Data availability after *R*^2^ test^*^**

|  | $\boldsymbol{R}^{\boldsymbol{2}}\boldsymbol{\leq\beta= 1}$ | $\boldsymbol{R}^{\boldsymbol{2}}\boldsymbol{\leq\beta= 0.95}$ | | $\boldsymbol{R}^{\boldsymbol{2}}\boldsymbol{\leq\beta= 0.9}$ | | $\boldsymbol{R}^{\boldsymbol{2}}\boldsymbol{\leq\beta= 0.87}$ | |
| --- | --- | --- | --- | --- | --- | --- | --- |
| **S1** | 5254 | 528 | 10.05% | 176 | 3.35% | 103 | 1.96% |
| **S2** | 5008 | 1212 | 24.20% | 729 | 14.56% | 571 | 11.40% |
| **S3** | 7008 | 1997 | 28.50% | 872 | 12.44% | 532 | 7.59% |
| **S4** | 6187 | 2347 | 37.93% | 1254 | 20.27% | 874 | 14.13% |
| **S5** | 5420 | 1804 | 33.28% | 926 | 17.08% | 604 | 11.14% |
| **S6** | 4998 | 2280 | 45.62% | 1497 | 29.95% | 1142 | 22.85% |
| **S7** | 1369 | 538 | 39.30% | 299 | 21.84% | 206 | 15.05% |
| **S8** | 4171 | 2034 | 48.77% | 1097 | 26.30% | 831 | 19.92% |
| **S9** | 4719 | 1697 | 35.96% | 856 | 18.14% | 601 | 12.74% |
| **S10** | 5037 | 1578 | 31.33% | 938 | 18.62% | 670 | 13.30% |

^*^ Columns 2, 3, 5, and 7 show the number of data samples in each dataset at different values of the *R*^2^ test threshold parameter, *β*. Columns 4, 6, and 8 show the percentage of samples remaining.

In the third step, all accepted current traces were low pass filtered (LPF). Two different LPFs were used, one for data classes S1−S9 with a cutoff frequency of 9 kHz and another for data class S10 with a cutoff frequency of 3 kHz. Since the sampling rate during the data acquisition phase for S10 was 10 kHz, compared to 30 kHz for S1−S9, all S10 current traces were linearly interpolated by a factor of 3 after low pass filtration.

Finally, all current traces were converted to conductance traces using eqn. below:

$Conductance =\frac{current \cdot{10}^{-8}}{V_{bias}} \left( G_{0} \right)$

where current is in units of 10 nA and *V_bias_* is the bias voltage in volts. The unit *G*_0_ in the above equation is the *conductance quantum* and is defined as follows: $G_{0}=\frac{2e^{2}}{h}=7.748\cdot{10}^{-5} S,$ where $e$ is the elementary charge and $h$ is Planck’s constant. For our datasets, the conductance values turn out to be in the range ${[10}^{-6.5}-{10}^{-0.5}] G_{0}.$

Figure S2 summarizes the sequence of pre-processing steps. Training vectors for our ML models are probability histograms computed from sample conductance traces. The efficacy of the pre-processing routine in smoothing out the histograms is demonstrated in Figure S3 which compares the conductance histograms for data class S1 computed from raw current traces vs. processed traces.

- 1. **Target labeling scheme for classification.**

Starting with the ten datasets (see Table 1 in the main article), we trained classifier models using two different target class labeling schemes. In the first scheme, unique DNA strands were assigned different class labels, irrespective of the voltage bias used for current measurement during SMBJ experiments. Since data types S2, S6, S7, S8, and S9 are of the same DNA strand, we end up with six target classes, [S1, {S2, S6, S7, S8, S9}, S3, S4, S5, S10], with this labeling scheme (called TLS-1). In the second scheme (called TLS-2), the datasets are assigned unique class labels based on the (strand, voltage bias) tuple. Although S2, S6, S7, S8, and S9 pertain to the same DNA strand, experimentations on S2 and S8 were conducted with a 10 *m*V bias, in contrast to 100 *m*V for S6 and S9 and 200 *m*V for S7 (see Table 1 in the main article). Based on the conductance histograms shown in Figure 1 in the main article, *prima facie* there appear to be enough differences induced by the choice of the bias voltage, to warrant consideration of a labeling scheme based on (strand type, bias voltage). With this scheme, we have eight target classes, [S1, {S2, S8}, S3, S4, S5, {S6, S9}, {S7}, S10].

1. **Data visualization.**

The *t*-stochastic neighbor embedding (*t*-SNE) method [1] is a powerful *local structure preserving* mapping method to visualize high dimensional data in two dimensions. A key parameter in *t*-SNE is ‘perplexity’, which controls the number of neighbors used by the algorithm to determine the local structure of a point. It is important to observe that not much should be made of the exact distance by which two distant points may be displayed on the low dimensional map generated by *t*-SNE since the algorithm is agnostic to ‘global distance information’ in the native feature space. An interesting discussion regarding the interpretability of *t*-SNE maps can be found in [2]. A recent application of the *t*-SNE algorithm to single cell transcriptomics can be found in [3]. Figure S4 shows the *t*-SNE visualizations of our ten 600-dimensional conductance probability histogram datasets (1000 per class) for *β* = 1, 0.95, and 0.87, based on a Barnes-Hut approximation with perplexity value = 30 and an initial 100-dimensional principal components analysis (PCA) projection. Overall, there appears to be a separation between the points in the different datasets. However, three important observations are in order.

First, the separation between S3 and S4, which differ by a single mismatch, appears to improve as *β* decreases. As far as these two classes are concerned, we should therefore expect a classifier to benefit from an aggressive *R*^2^ test-based pre-filtering of the data.

Second, the choice of bias voltage matters. This is evident from an examination of the clusters depicted by (S2,S8), (S6,S9), and S7. Although these datasets correspond to the same strand, we see from Table 1 in the main article that the bias voltage is 10 *m*V for (S2,S8), 100 *m*V for (S6,S9), and 200 *m*V for S7. Experiments conducted with different bias voltages on the same molecule can induce very different conductance properties, which is evident in the histograms in Figure 1 in the main article as well as in the natural separation we observe between (S2,S8), (S6,S9) and S7 in Figure S4. This lends some empirical evidence that a target class labeling scheme based on a (strand, bias voltage) tuple may be a more prudent choice than a purely strand based labeling scheme which may result in a diffuse cluster of identically labeled points occupying a wide swathe in the feature space, which can be detrimental to an ML based classification approach.

Third, we observe that the S10 cloud map ‘straddles’ the (S2,S8) cloud considerably and no amount of *R*^2^ filtering appears to ameliorate this issue, unlike S3 and S4. In fact, without the aid of class labels, it would be natural to expect one cluster formed by S2, S8, and S10. It is interesting to note that our first and third observations regarding (S3,S4) and (S2,S8,S10) are also validated by classical multidimensional scaling [4] projection plots (see Figure S5).

1. **Analysis of secondary binary classifier for S3 and S4.**

In the main article, we have established that a single XGboost classifier works phenomenally well in classifying the ten datasets into six (strand based) classes or eight (strand, bias voltage based) classes, with the exception of S3 and S4 which differ by a single mismatch. Nevertheless, based on our observations in conjunction with Figure S4, we recommend that experimental data on the same strand but with different bias voltages be coded as different classes. We have also demonstrated the challenges in designing a monolithic classifier with one set of values for (*N_bins_*, *β*, *H*) which works equally well for all data classes. An alternate architectural option might be to employ a 2-stage tandem classifier where S3 and S4 are combined as one target class, say superclass 3_4, in the first stage primary classifier, followed by a second stage binary classifier operating on S3 and S4 with a lower value of *β* and other parameters, suitably tuned for these classes*.* In this option, the second stage classifier is invoked if the prediction from the first stage indicates a ‘class 3_4’. Another architectural option might be to design the primary classifier with 6 or 8 classes (S3 and S4 coded as different classes) and pair it with a secondary binary classifier operating on S3 and S4 for additional verification. If both classifiers predict the same class, that should obviously enhance the likelihood of correct identification. We believe that this tandem classifier approach, although motivated in this paper by our need to reject invalid experimental observations (driven by the *R*^2^ test threshold, *β*), will be useful to distinguish between strands with similar conductance distributions, even if uncertainties associated with experimental conditions are rendered moot. In this section, we discuss the performance of a secondary binary classifier operating on S3 and S4 only.

Often, the performance of an unsupervised clustering algorithm on a labeled database is used as a benchmark for classifier performance. Toward that goal, we report the performance of a baseline spectral clustering algorithm on S3 and S4, using *N_bins_* = 600 and *H* = 30. Given a set of *n* points, $\{x_{i} :1\leq i\leq n\}$, in *d*-dimensional space, the steps involved in a spectral clustering [5] approach are as follows: (i) construct a similarity graph on the *n* points, (ii) perform an eigen-decomposition of the Laplacian matrix of the similarity graph, and (iii) use a clustering method such as *k*-means to cluster the points using the *p* (*p* ≤ *d*), eigenvectors of the graph Laplacian corresponding to the *p* smallest eigenvalues. The similarity between points *x_i_* and *x_j_* in the similarity graph is computed as follows:

$s_{ij}= \exp\left( -\left\| x_{i}-x_{j} \right\|^{2}/ \sigma^{2} \right)$

where *σ* is the scale parameter. Several methods exist for constructing the similarity graph; we use the nearest neighbor graph construct with a specified number of neighbors for each point. In addition, several forms of the Laplacian matrix are possible; we use the symmetric normalized Laplacian matrix defined in [6]. Figure S6 shows the class accuracies as a function of the number of neighbors, for *p* = 2, *σ* = 1 and *β* = 1, 0.95, and 0.87 (these simulations were conducted in MATLAB). Generally speaking, the clustering quality can be sensitive to the choice of *σ*. For our datasets, however, we found that the solution is robust to $\sigma\in[0.5, 2]$. Representative benchmark accuracy indices for S3 and S4 are (i) [82.9, 90.8] % for *β* = 1, (ii) [99.2, 91.3] % for *β* = 0.95, and (iii) [100, 99.1] % for *β* = 0.87, all numbers corresponding to 20-nearest neighbor similarity graphs. We observe that the spectral clustering method works remarkably well for both classes at *β* = 0.87. At *β* = 0.95, the accuracy of S3 is almost 8% better than the accuracy of S4, which, quite interestingly, is reversed at *β* = 1. While some degree of explanation of this behavior can be offered from a graph cut viewpoint of the spectral clustering method, possibly applied to a low dimensional projection of the data, it is not necessary for the context of this article.

We now discuss the performance of a secondary binary support vector machine (SVM) classifier trained with 700/300 training/testing histograms per class. Our choice of SVM as the classification algorithm was driven primarily by the clean separation which appears to exist between two-dimensional projections of the two classes at *β* = 0.87, as is evident from the *t*-SNE and classical multidimensional scaling (MDS) [4] visualizations in Figures S4 and S5. Simulations were conducted in MATLAB using a vanilla SVM (linear kernel) with a regularization parameter of *C* = 1. The matrix of training histograms was standardized on a per bin basis. While we did not find any evidence that standardization enhances XGboost’s performance, the binary SVM classifier benefitted immensely from standardized data. Viewing the mismatched sample, S4, as the positive class and S3 as the negative class, training with unstandardized data resulted in substantially lower sensitivities but relatively high specificities, which is not desirable if detection of a mutant strain is the primary objective, even at the expense of a slightly higher false positive rate. See Figure S7 for details.

Table S2 presents the classification accuracies with standardized data as a function of *H* and *β*, averaged over 100 runs, for *N_bins_* = 600 and 60. First, we observe that class accuracies reach almost 100% for (*β* = 0.87, *H* ≥ 30) and coarse binned histograms work almost as well as fine grained histograms. The only cases where coarse histograms are decidedly better than fine histograms are when (*β* = 1, *H* ≤ 20), but these aren’t of practical interest due to rather low class accuracies. Second, the class accuracies drop significantly as *β* increases, which is consistent with the behavior of the primary XGboost classifier. At *H* = 30, the drop is almost 7-8%, comparable to what we saw in Figures 4(a) and 4(b) in the main article. Third, at baseline values of (*β* = 0.95, *N_bins_* = 600, *H* = 30), class accuracies are [99.45, 99.57] %, which are almost 2.5-3% better than the accuracies indicated in Figure 3 in the main article for the primary classifier. This demonstrates the benefit of using a tandem classifier approach compared to a monolithic model.

**Table S2. Class accuracies in percentages for a secondary binary SVM classifier operating on S3 and S4^*^**

| *H* | *β* = 1 | | *β* = 0.95 | | *β* = 0.87 | |  | *H* | *β* = 1 | | *β* = 0.95 | | *β* = 0.87 | |
| --- | --- | --- | --- | --- | --- | --- | --- | --- | --- | --- | --- | --- | --- | --- |
|  | S3 | S4 | S3 | S4 | S3 | S4 |  |  | S3 | S4 | S3 | S4 | S3 | S4 |
| 10 | 76.53 | 76.73 | 91.33 | 90.54 | 98.41 | 97.82 |  | 10 | 81.34 | 78.03 | 91.57 | 89.52 | 96.07 | 94.76 |
| 20 | 85.97 | 86.17 | 98.48 | 97.46 | 99.88 | 99.39 |  | 20 | 89.69 | 88.78 | 96.54 | 95.94 | 98.53 | 98.56 |
| 30 | 92.36 | 92.67 | 99.45 | 99.57 | 100.00 | 99.89 |  | 30 | 92.87 | 92.53 | 98.42 | 98.23 | 99.60 | 99.65 |
| 40 | 95.33 | 95.12 | 99.80 | 99.13 | 100.00 | 100.00 |  | 40 | 95.03 | 95.12 | 98.86 | 98.94 | 100.00 | 99.96 |
| 50 | 97.08 | 97.05 | 99.82 | 99.99 | 100.00 | 100.00 |  | 50 | 96.43 | 96.69 | 99.42 | 99.36 | 100.00 | 100.00 |

^*^ The left and right tables correspond to *N_bins_* = 600 and *N_bins_* = 60 respectively. The accented rows and columns correspond to baseline parameter values (*β* = 0.95, *N_bins_* = 600, *H* = 30). For comparison, the (S3,S4) class accuracies obtained from spectral clustering with (*N_bins_* = 600, *H* = 30) are (i) [82.9, 90.8] % for *β* = 1, (ii) [99.2, 91.3] % for *β* = 0.95, and (iii) [100, 99.1] % for *β* = 0.87.


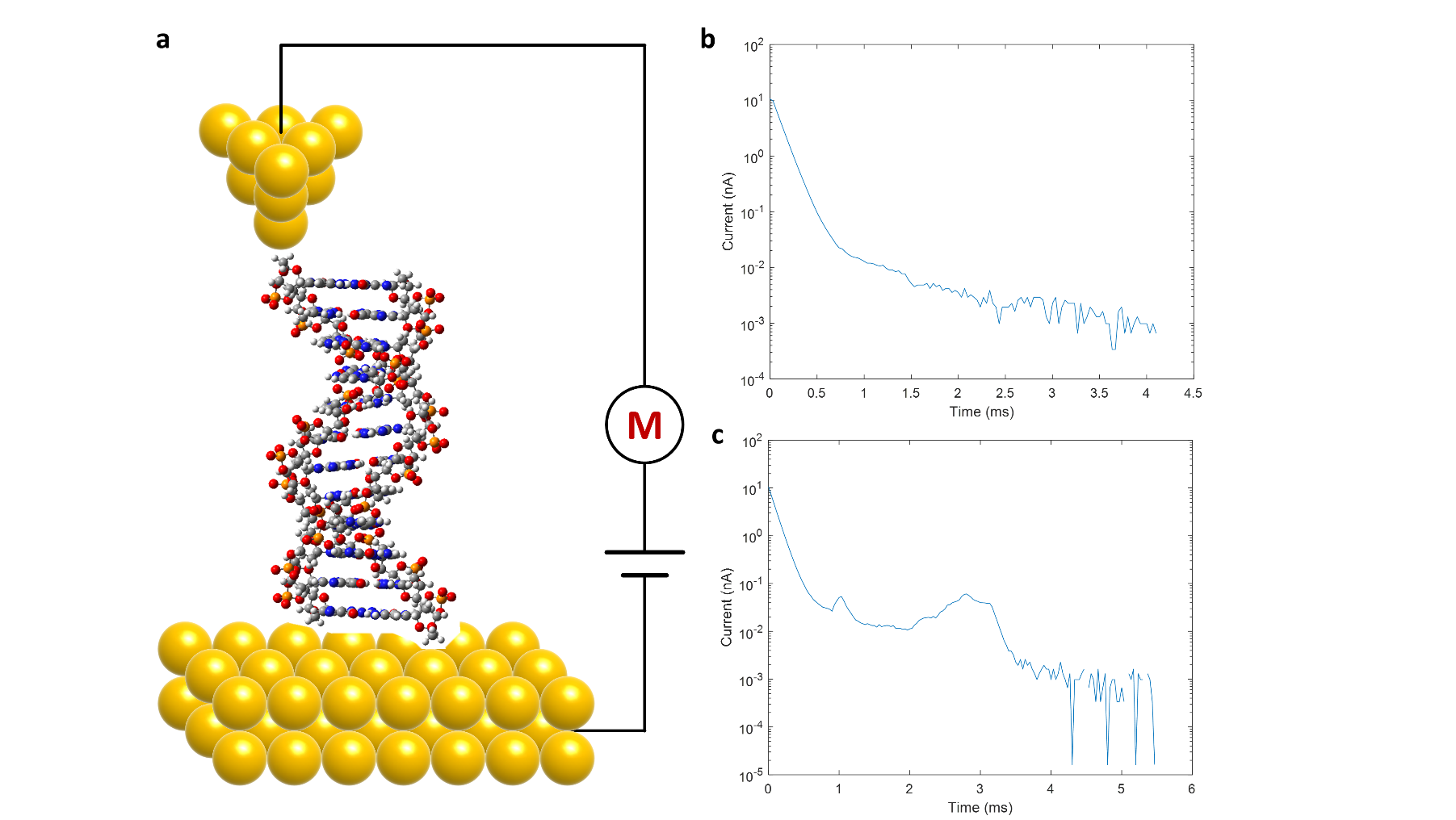


**Figure S1. Raw experimental input data.** (a) An idealized schematic of the single-molecule break junction (SMBJ) approach for conductance measurement. The measurement setup includes a bias voltage and a preamplifier [7]. (b) Sample current trace with no DNA binding between the gold electrode and the substrate. (c) Sample current trace with DNA binding between the gold electrode and the substrate.


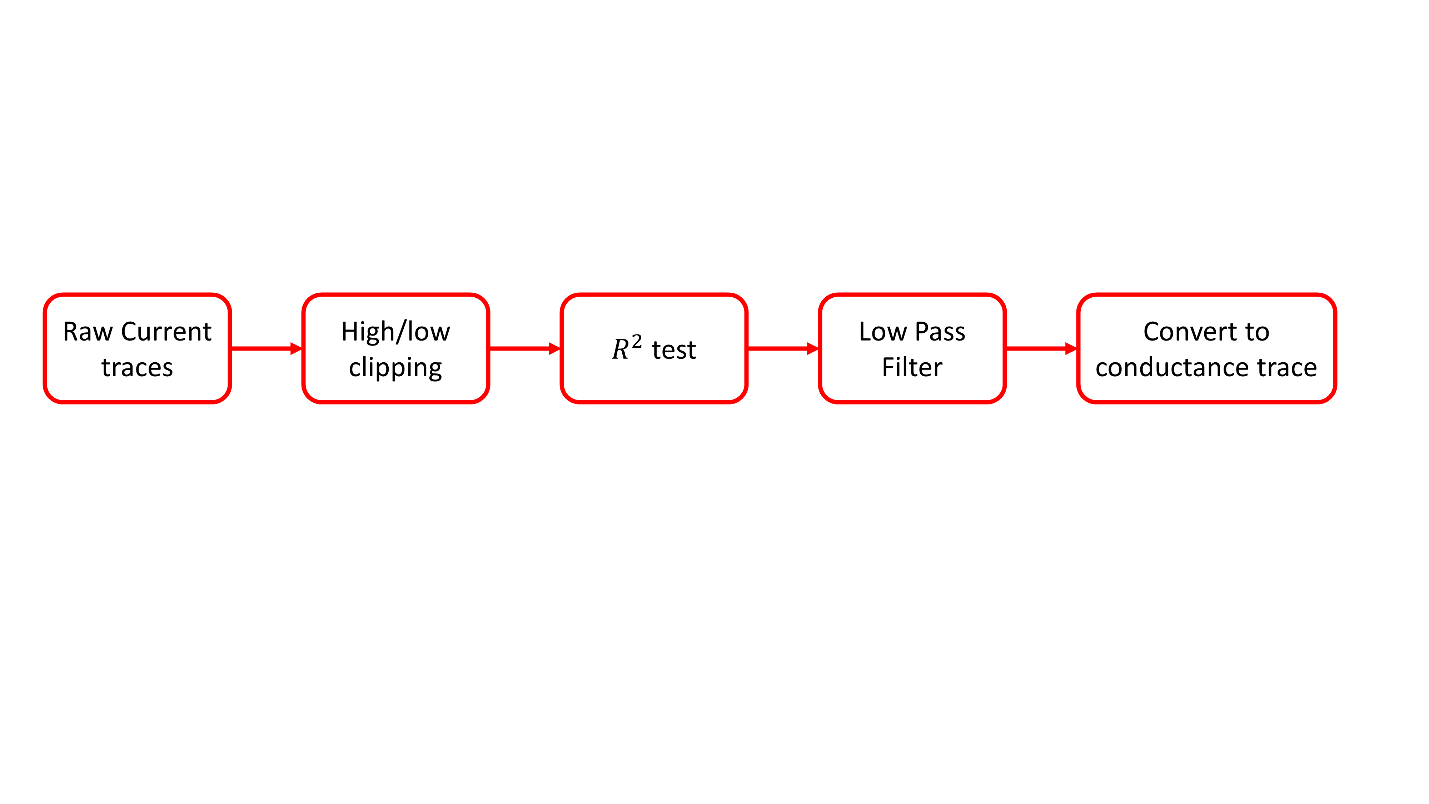


**Figure S2. Sequence of pre-processing steps for converting raw current traces to conductance traces.**


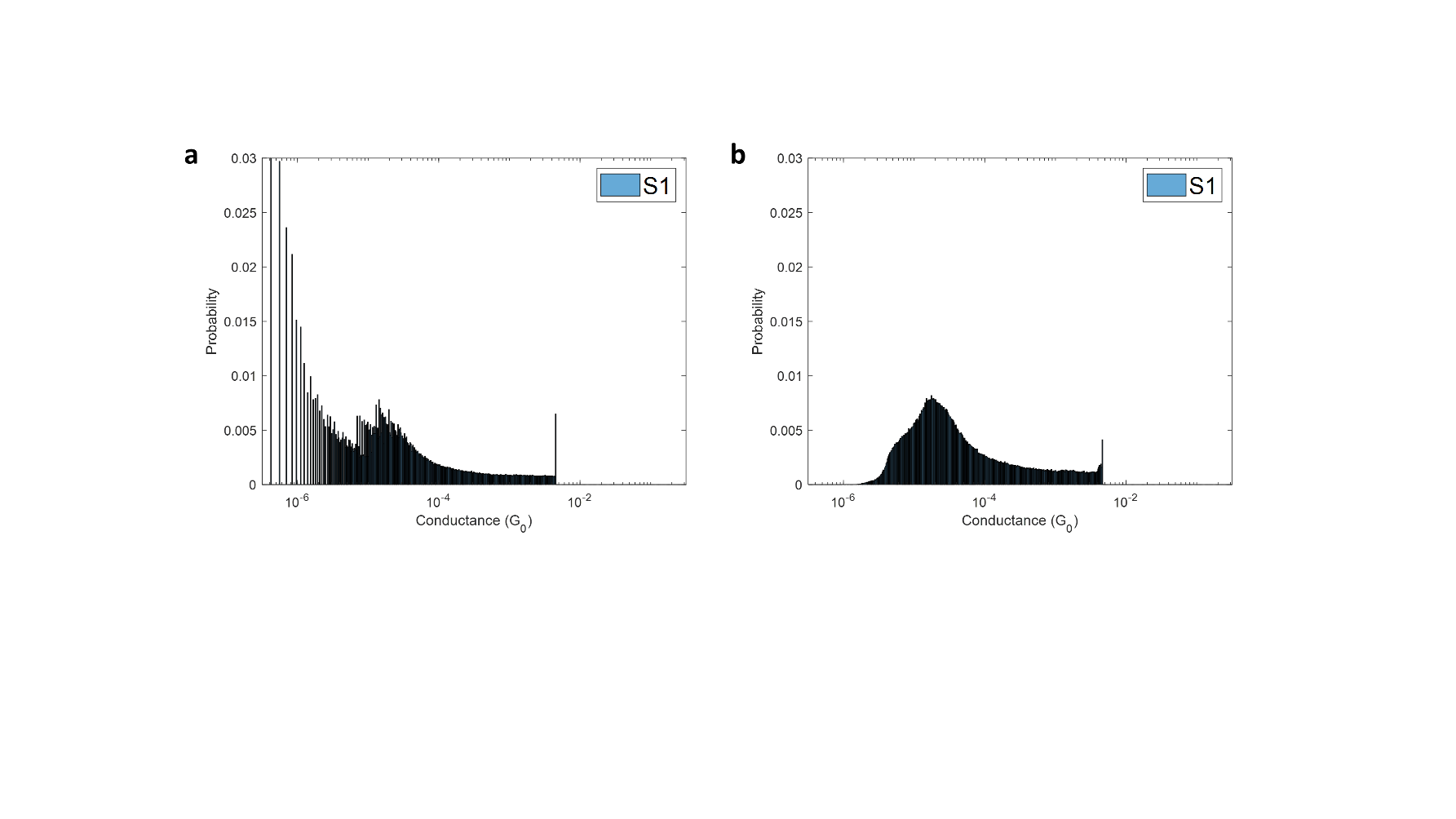


**Figure S3. Representative large sample conductance histograms for data class S1 using threshold parameter *β* = 1.** (a) Derived from raw current traces. (b) Derived from processed current traces.


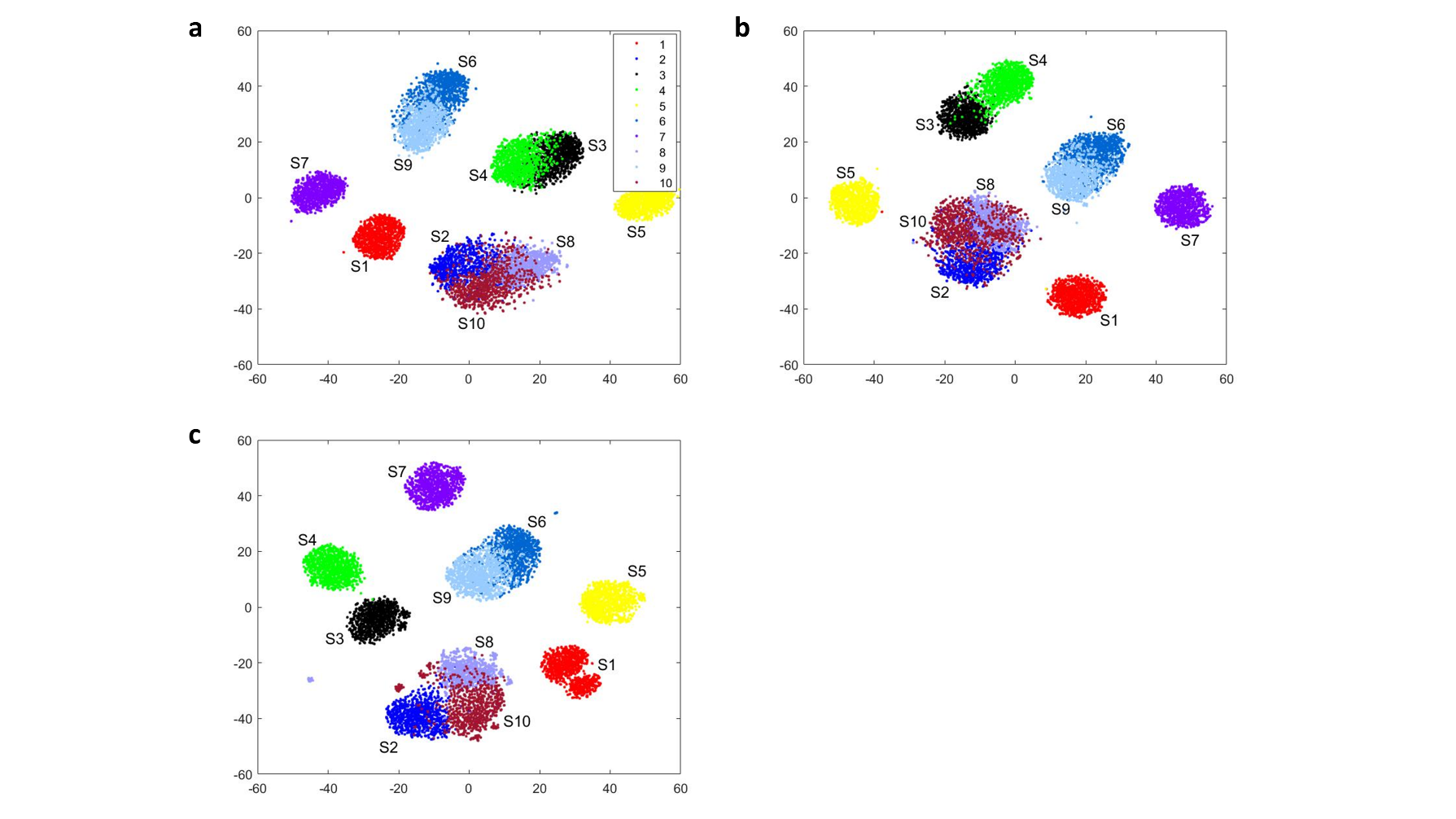


**Figure S4. *t*-SNE visualization of our datasets.** (a) *R*^2^ ≤ *β* = 1, (b) *R*^2^ ≤ *β* = 0.95, and (c) *R*^2^ ≤ *β* = 0.87. We have adopted the same ‘bluish’ color scheme for S2, S6, S7, S8, and S9 since they correspond to the same molecule, though with three different biases.


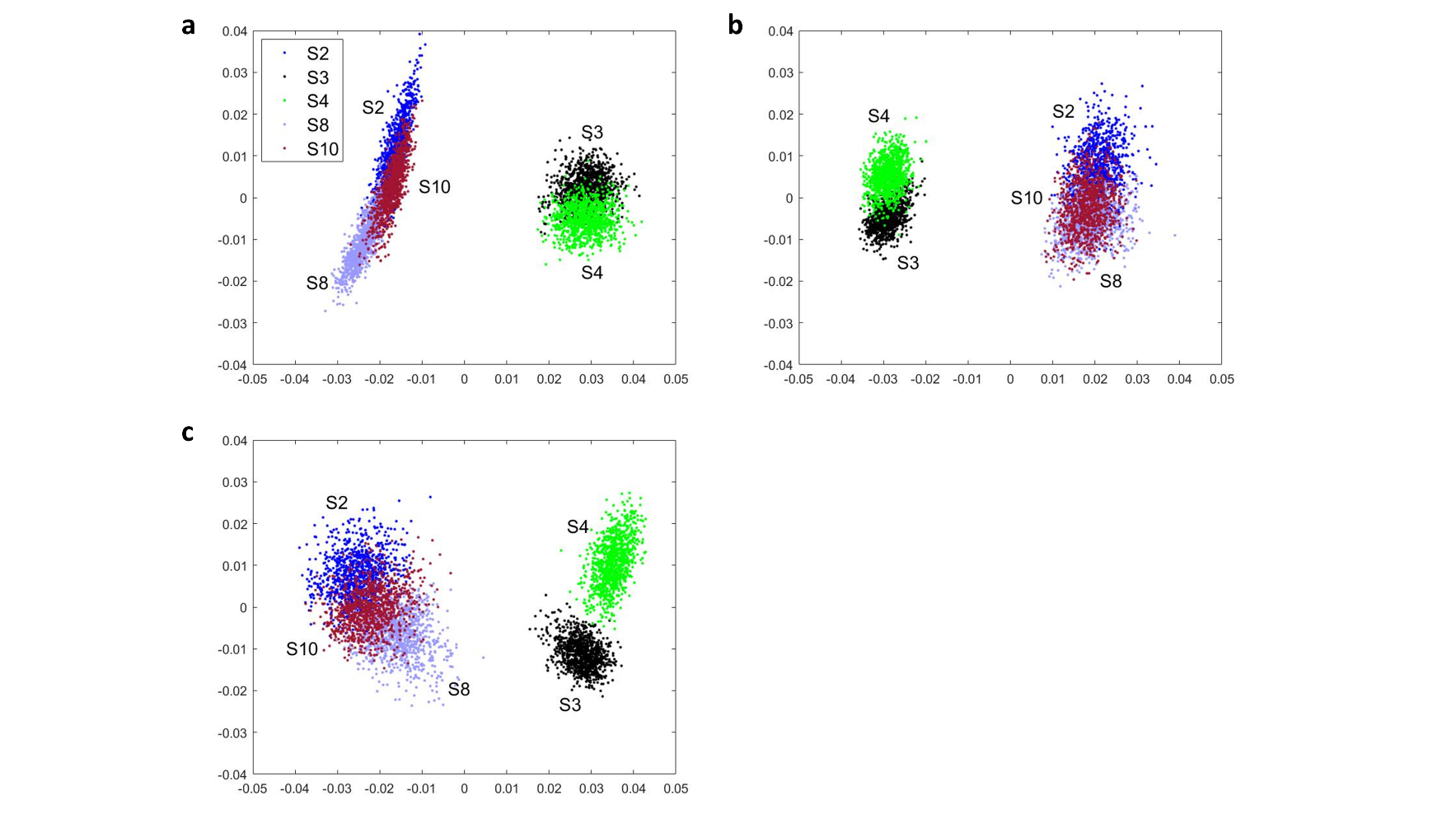


**Figure S5. Classical multidimensional scaling (MDS) visualizations.** MDS visualizations of S2, S3, S4, S8, and S10 datasets for (a) *R*^2^ ≤ *β* = 1, (b) *R*^2^ ≤ *β* = 0.95, and (c) *R*^2^ ≤ *β* = 0.87.


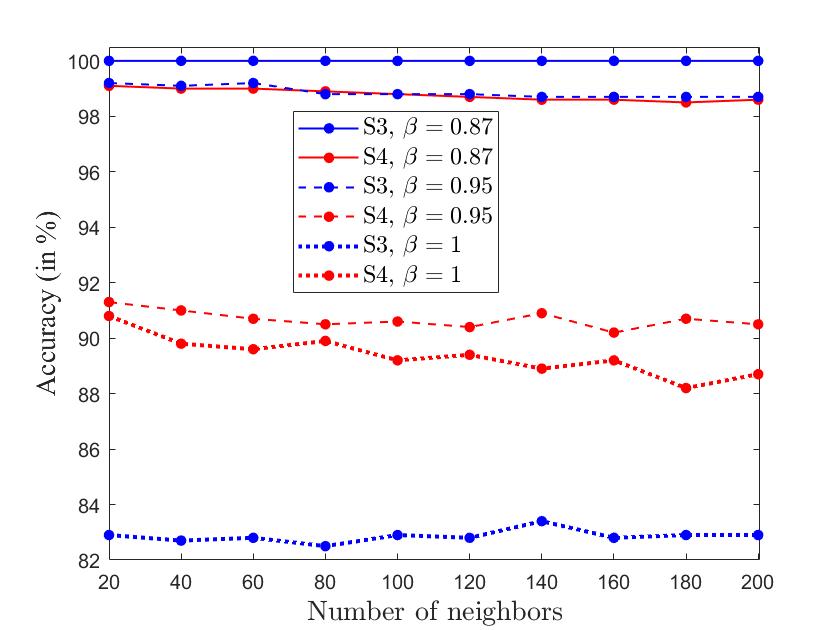


**Figure S6. Clustering of S3 and S4 data.** Class accuracies obtained from spectral clustering of S3 and S4 conductance histogram data (1000 per class) as a function of the number of neighbors used to construct a similarity graph on the data, using *N_bins_* = 600 and *H* = 30.


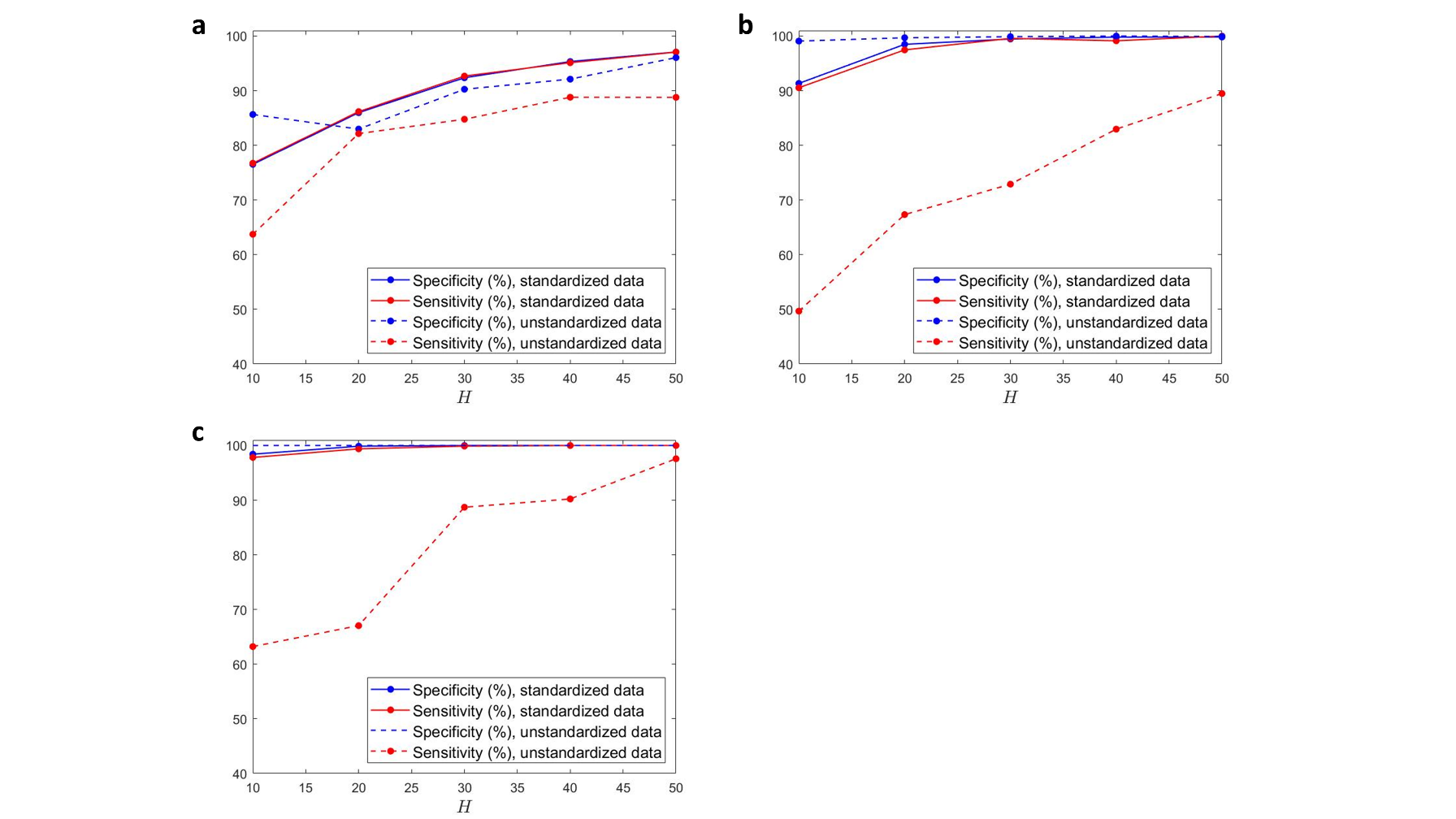


**Figure S7. Binary SVM classifier on S3 and S4.** We view the mismatched sample, S4, as the positive sample and S3 as the negative sample. This figure illustrates the impact of data standardization on the sensitivity and specificity of a binary vanilla SVM classifier (linear kernel with regularization parameter *C* = 1) operating on S3 and S4 with (a) *R*^2^ ≤ *β* = 1, (b) *R*^2^ ≤ *β* = 0.95, and (c) *R*^2^ ≤ *β* = 0.87. When the data is standardized, the classifier is naturally balanced which leads to approximately equal sensitivities and specificities. When the data is not standardized, we end up with a rather low sensitivity but high specificity classifier. While it is feasible to tune the classifier balance with unstandardized data, the primary objective of this figure is to illustrate the tremendous impact of standardization on the same baseline classifier.


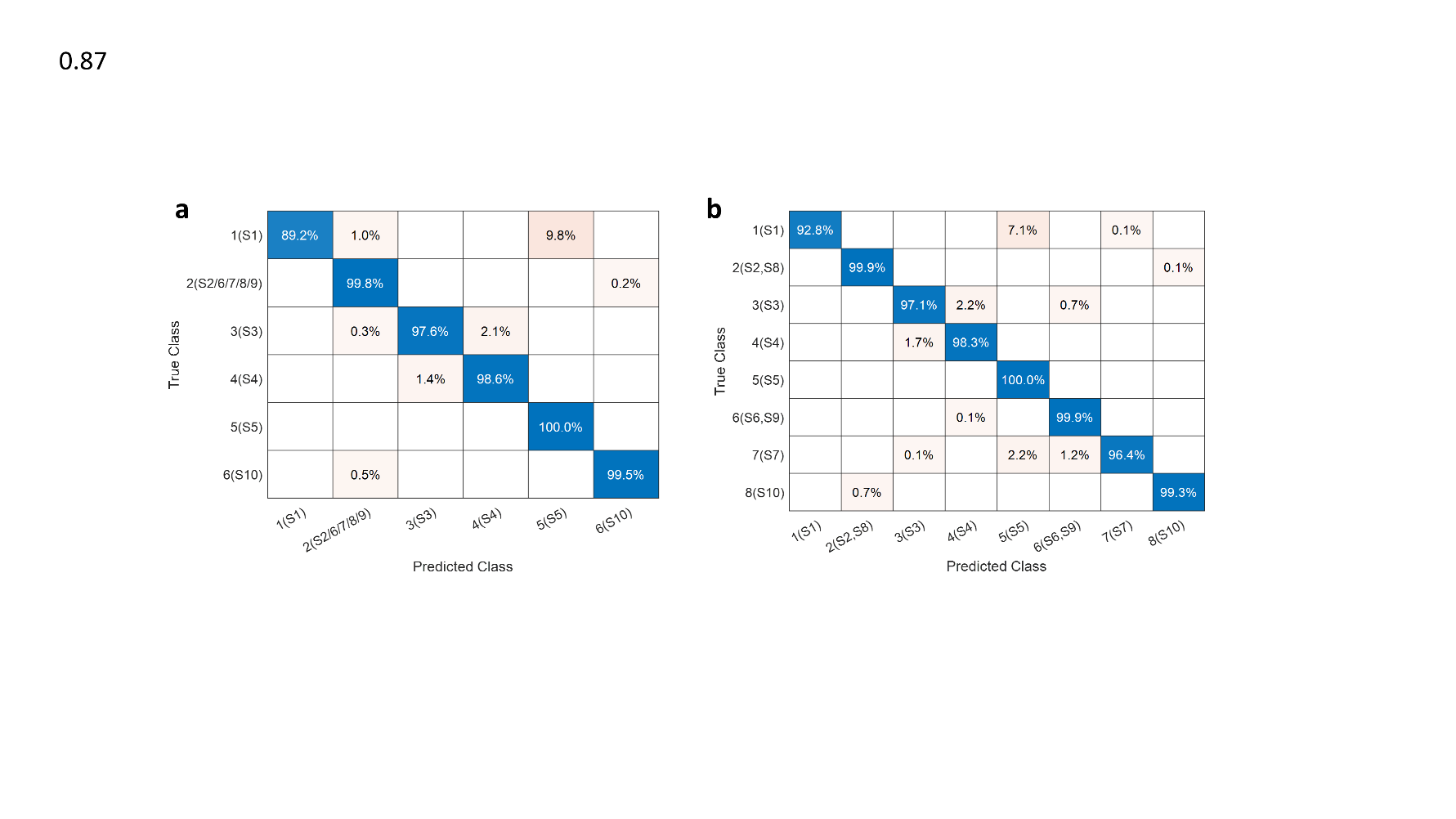

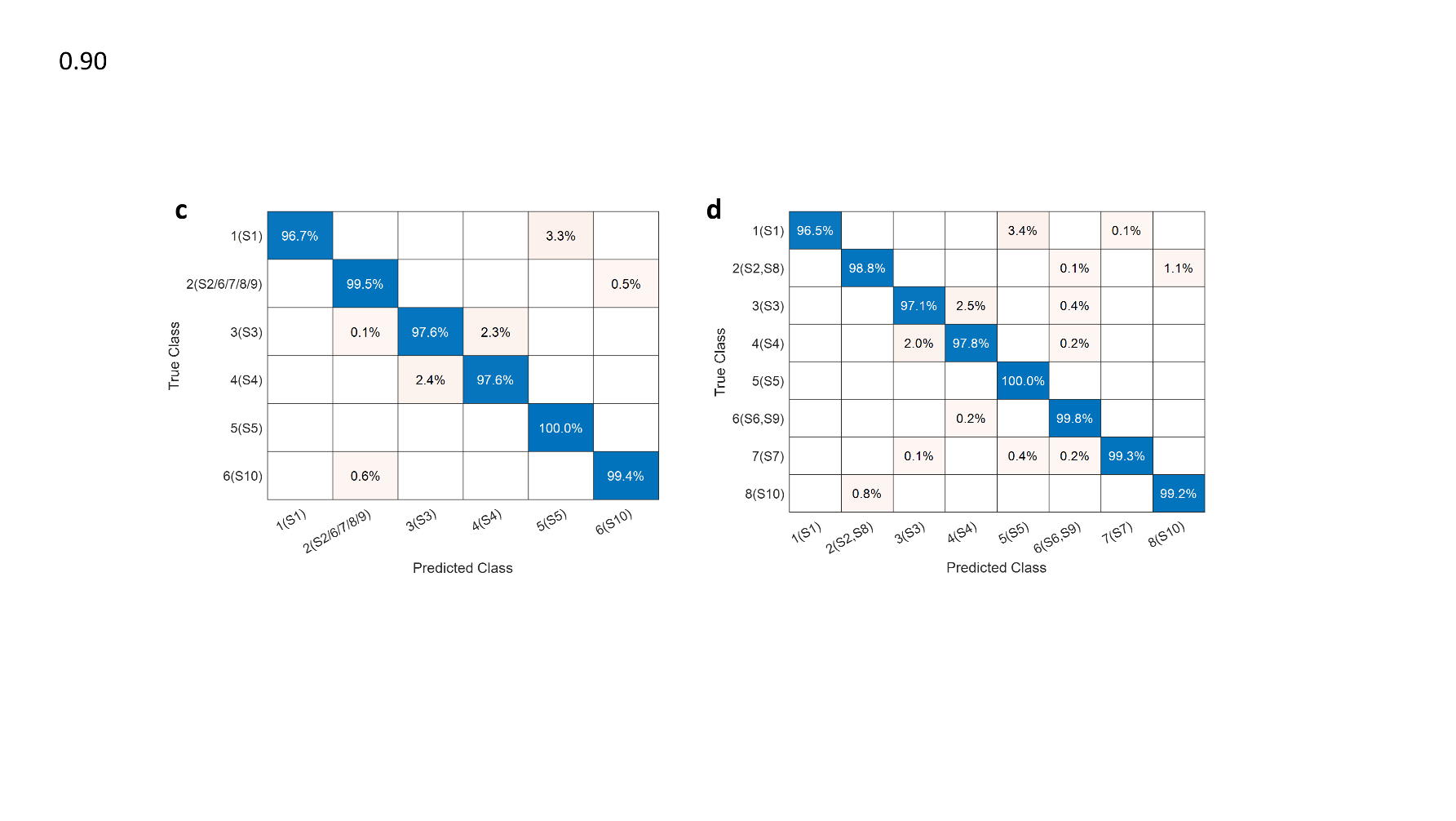

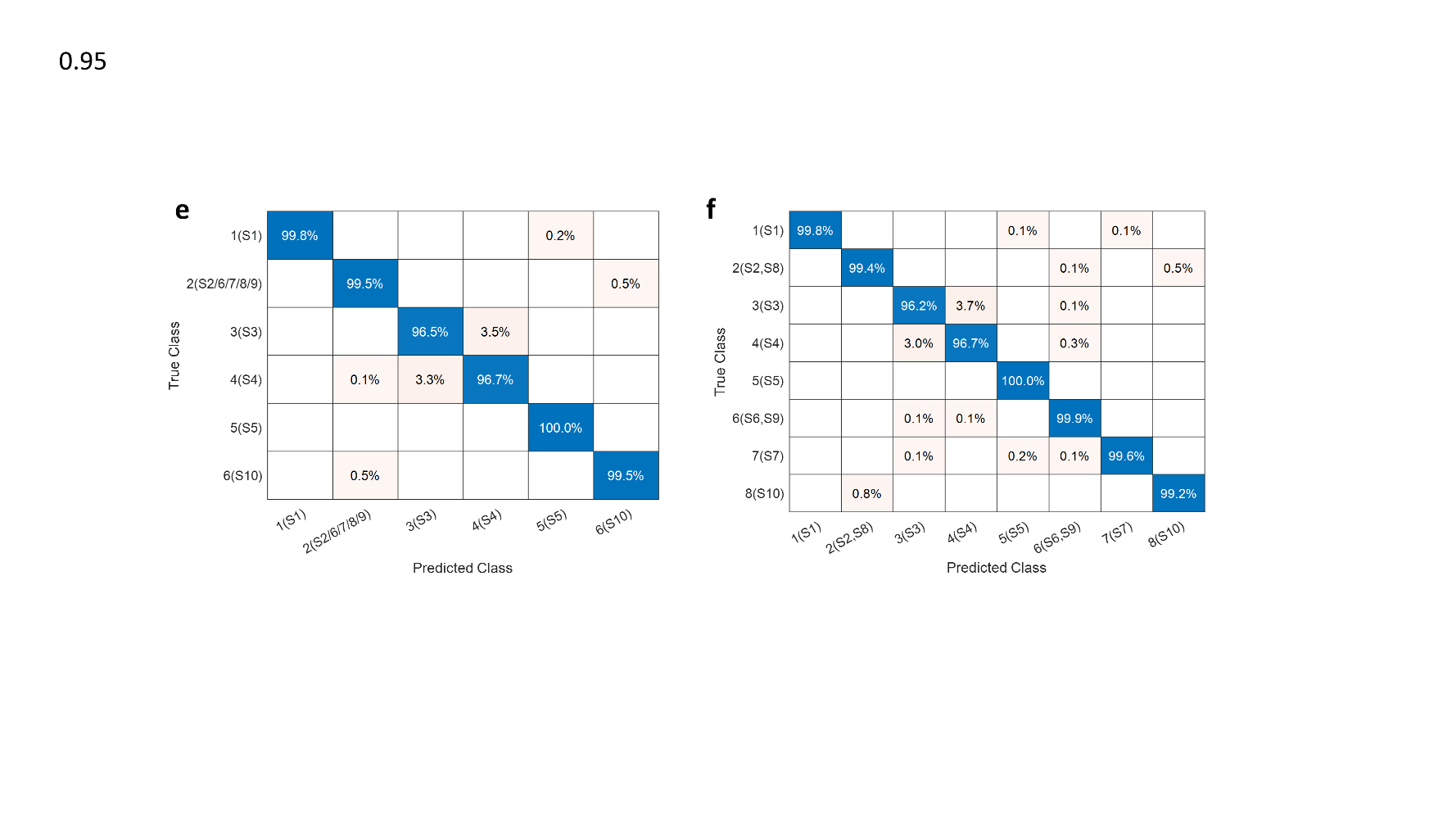

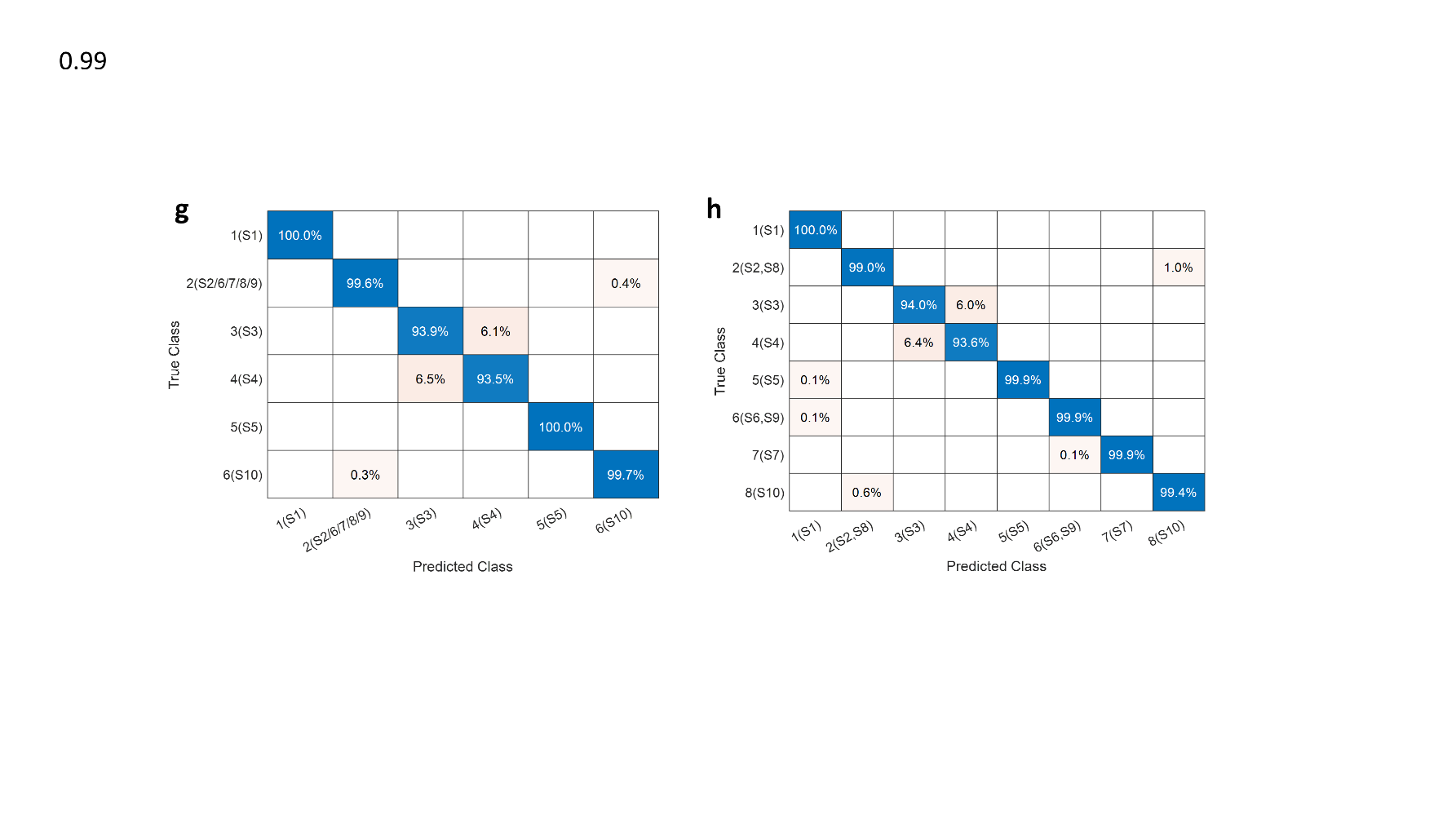

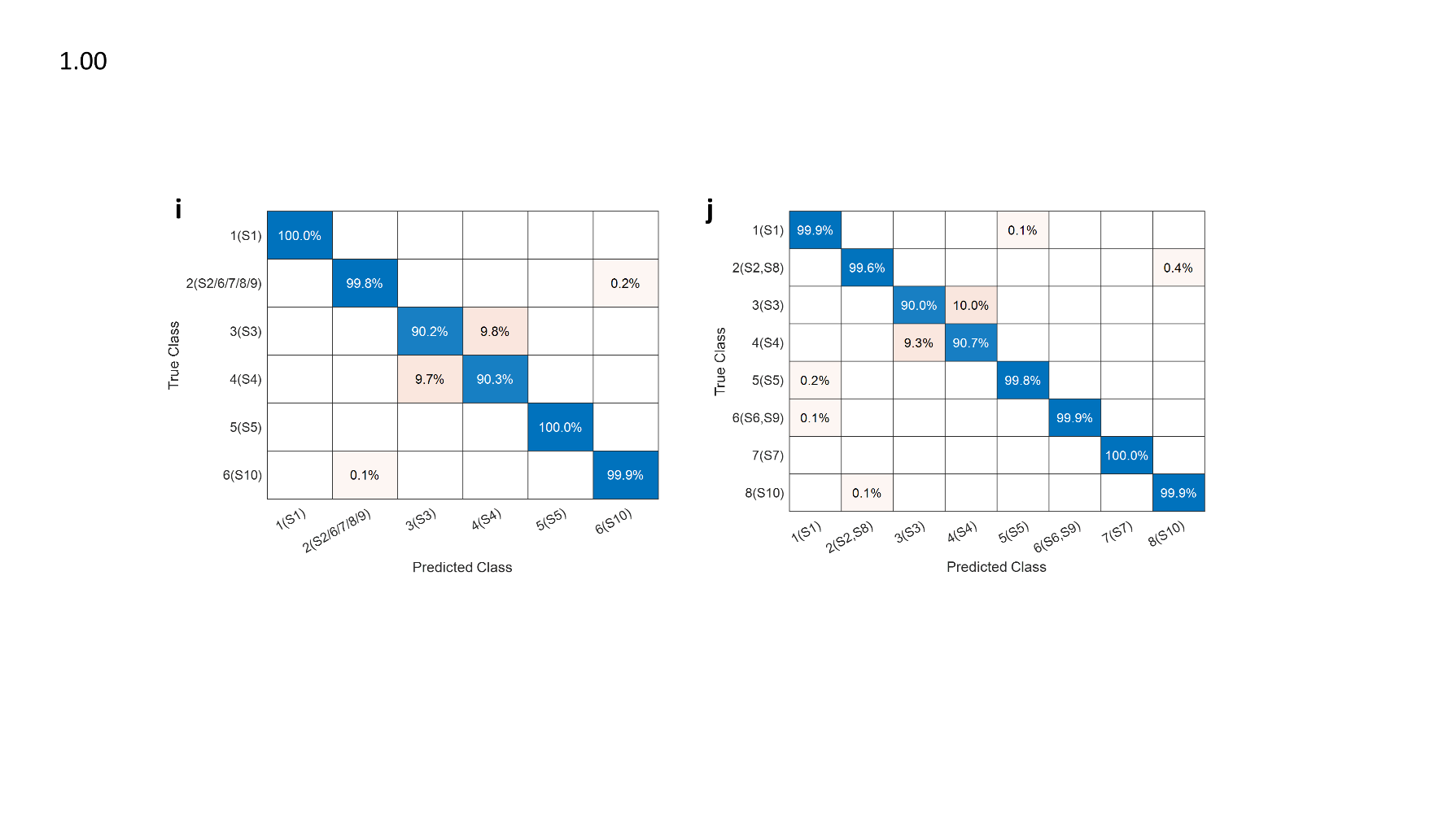


**Figure S8.** **Performance analysis of baseline classifiers with respect to the *R*^2^ test threshold parameter, *β .*** The left-column and right-column figures correspond to TLS-1 with 6 classes and TLS-2 with 8 classes respectively. The confusion matrices correspond to baseline parameter values (N_bins_ = 600, H = 30) and changing parameter *β.* (a), (b) for *β* = 0.87. (c), (d) for *β* = 0.90. (e), (f) for *β* = 0.95 (same as Figure 3 in the main article). (g), (h) for *β* = 0.99. (i), (j) for *β* = 1.00


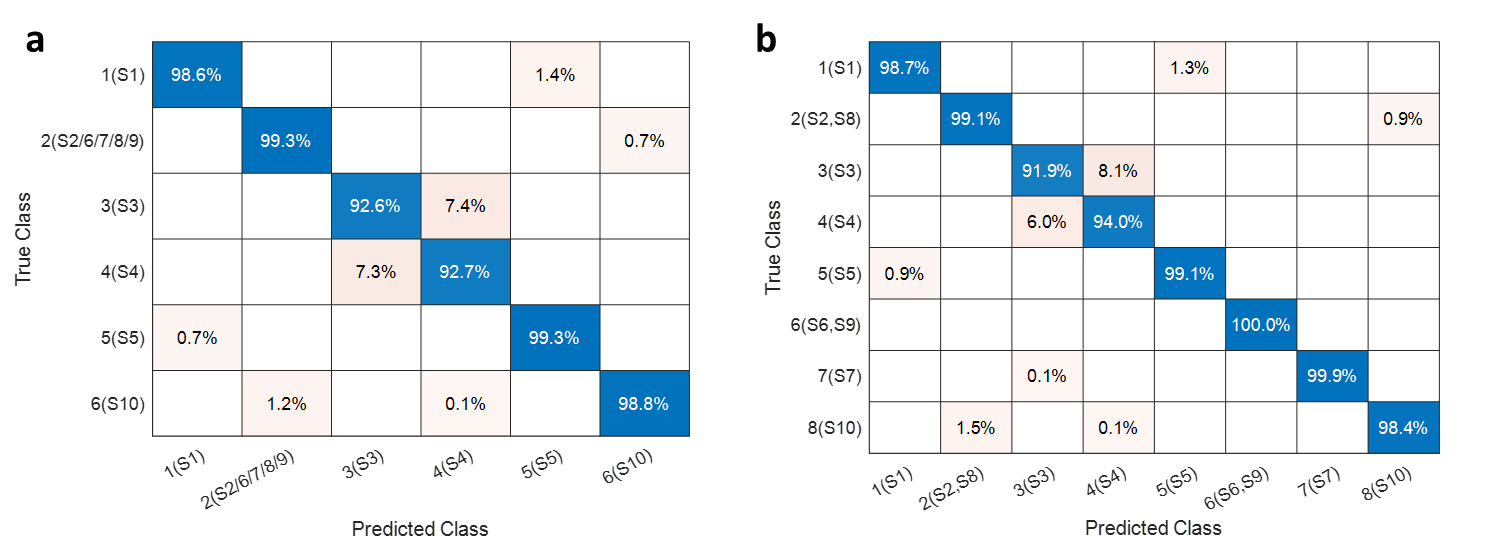

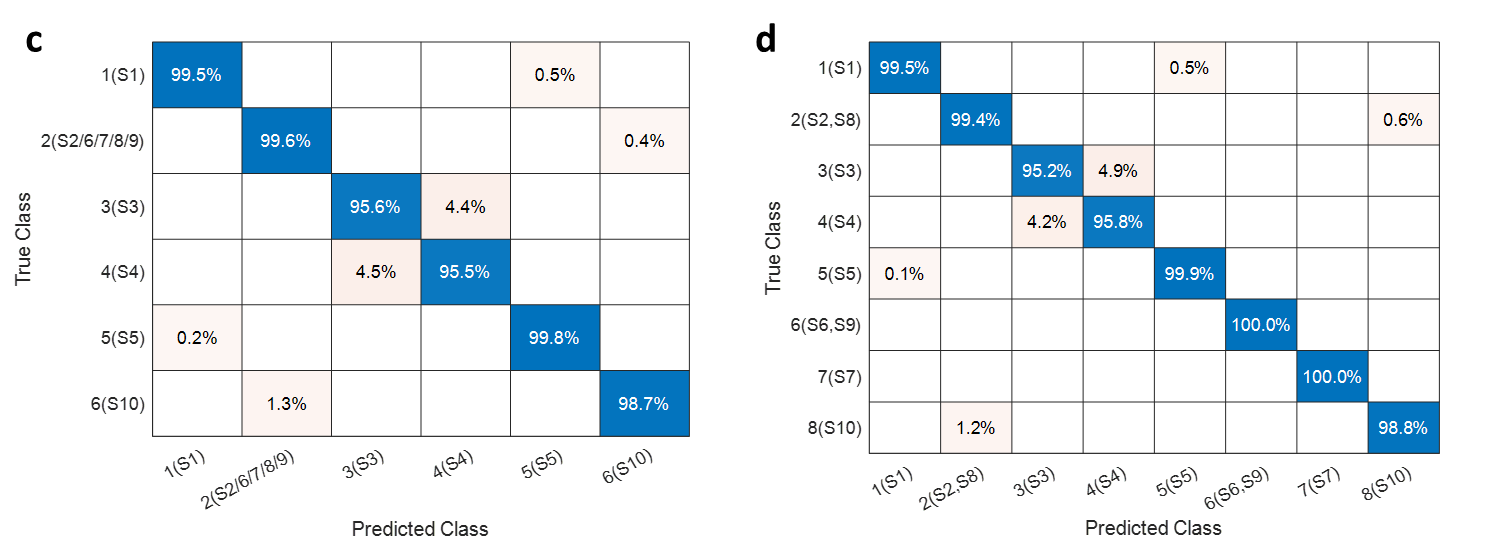

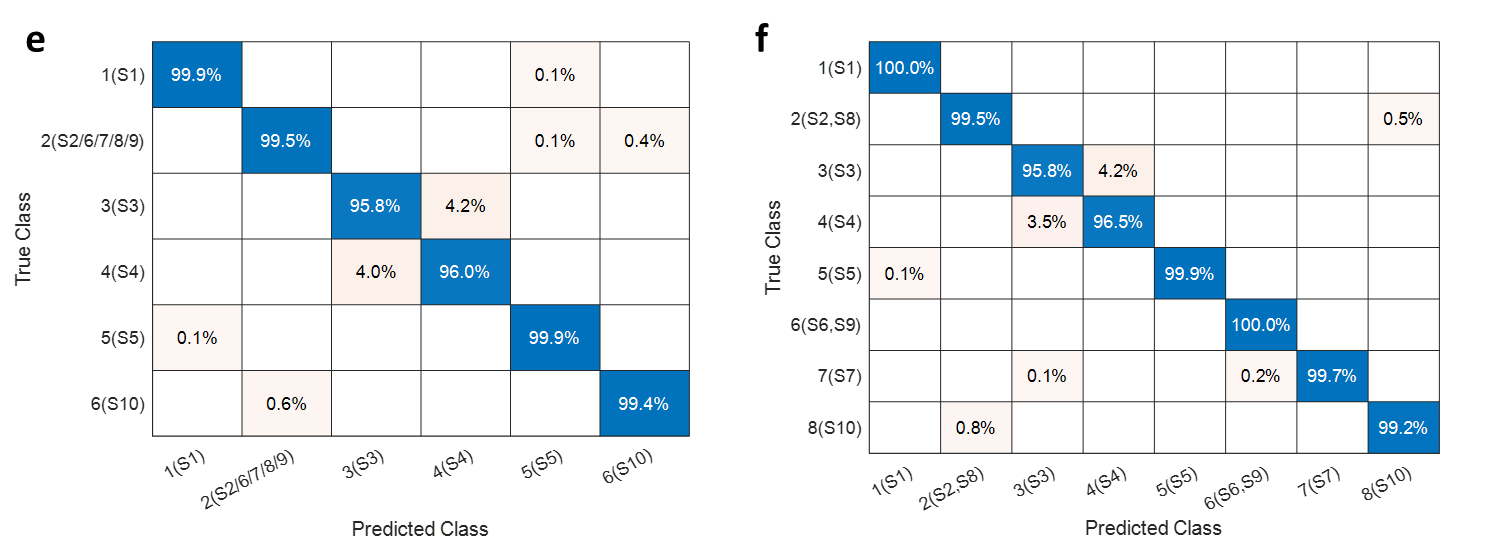

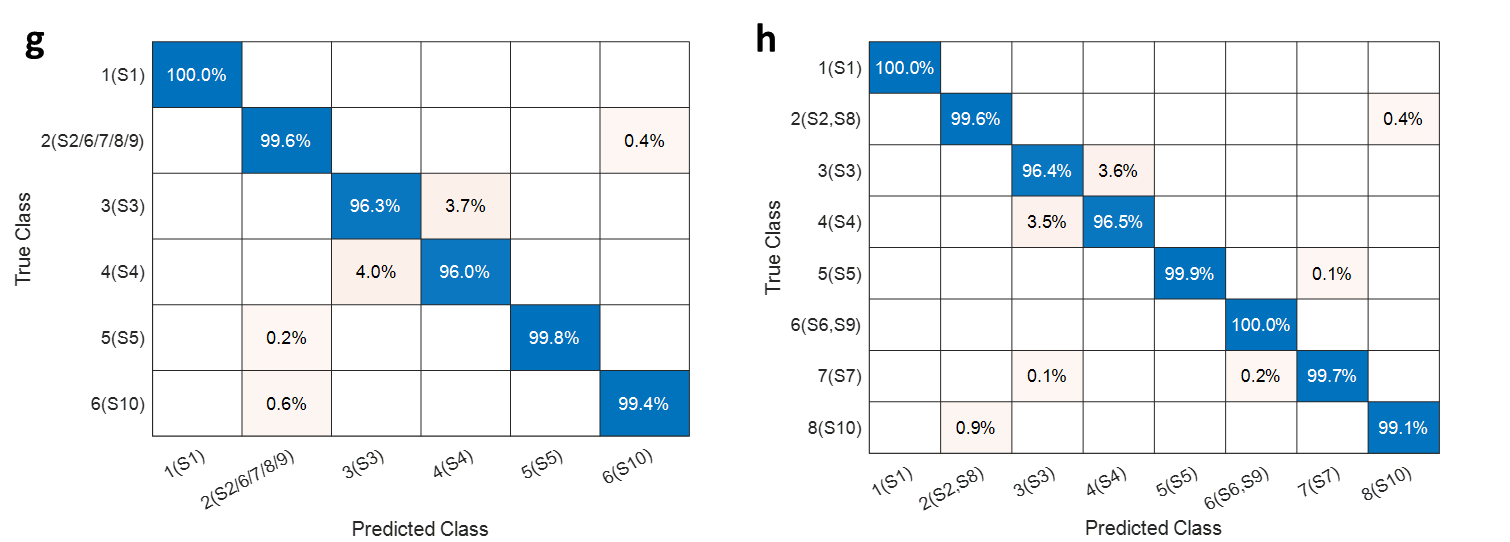

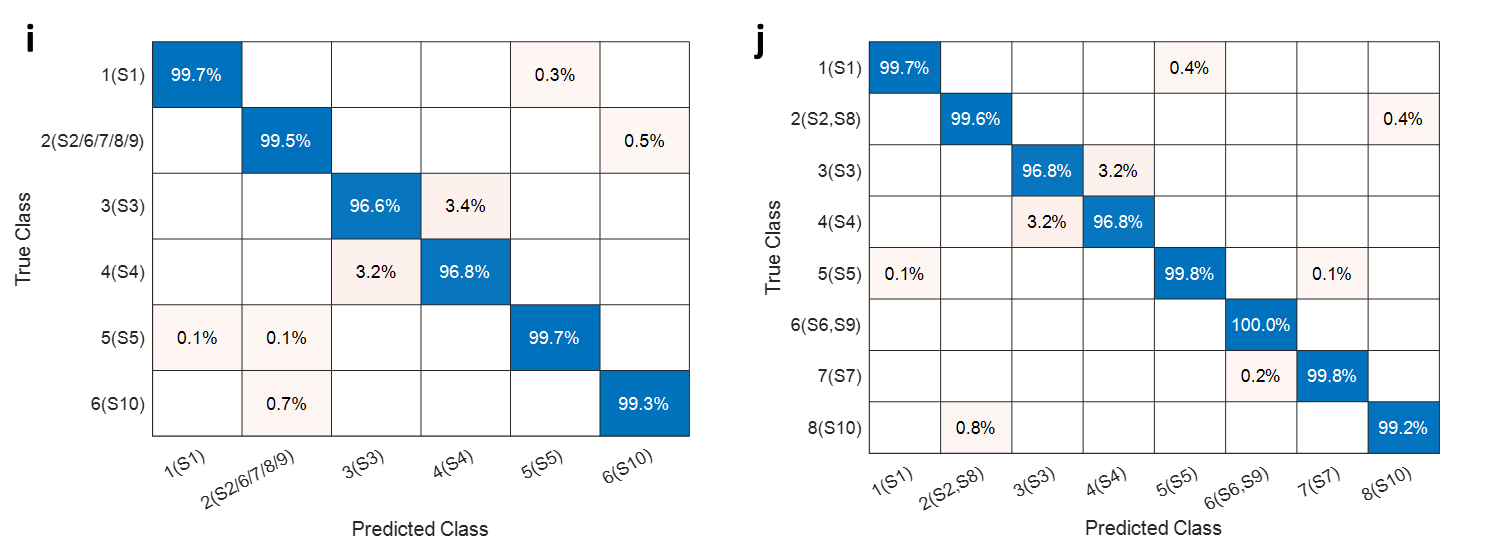

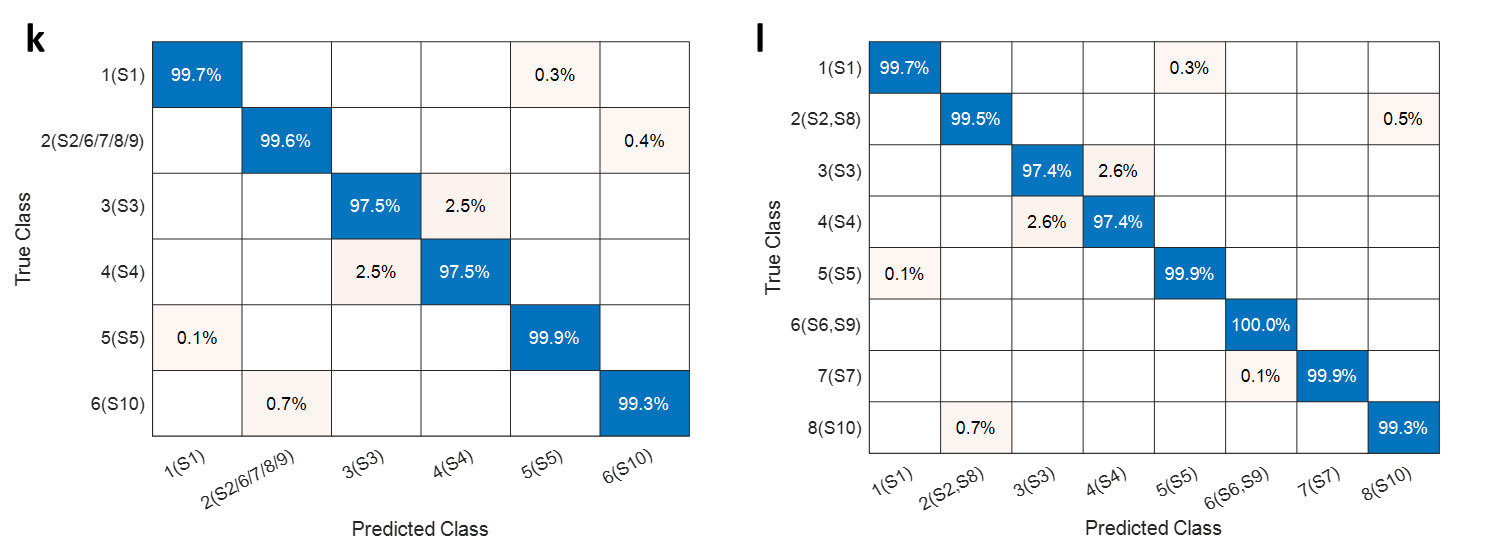

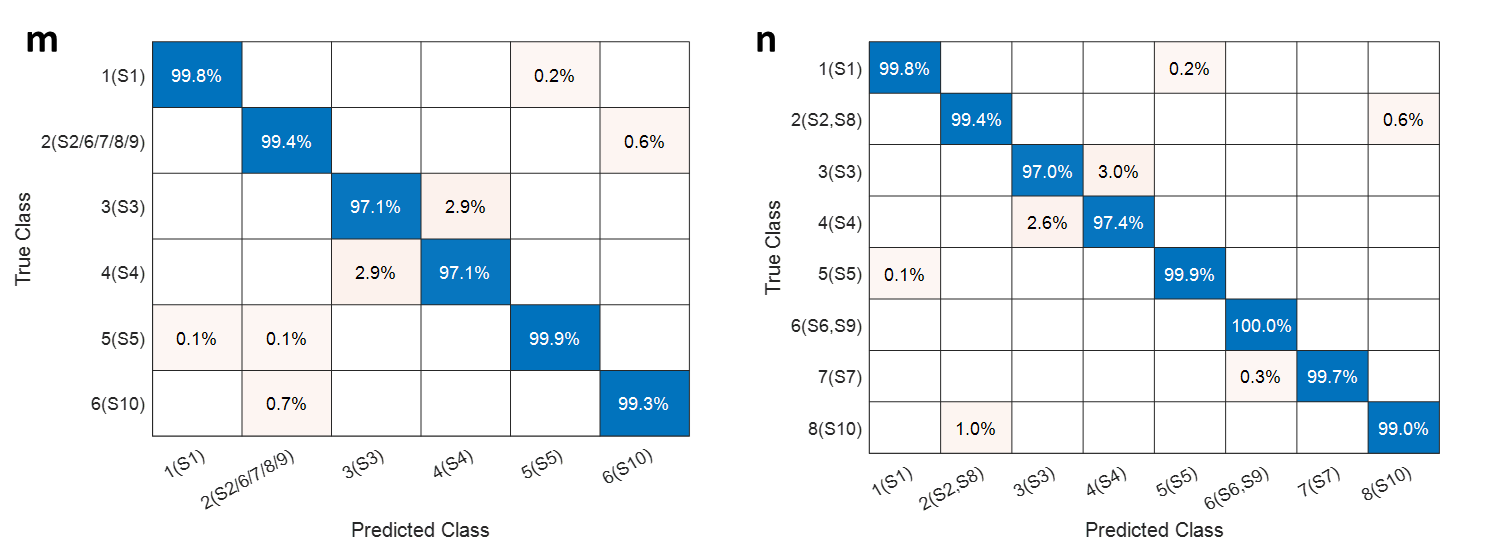

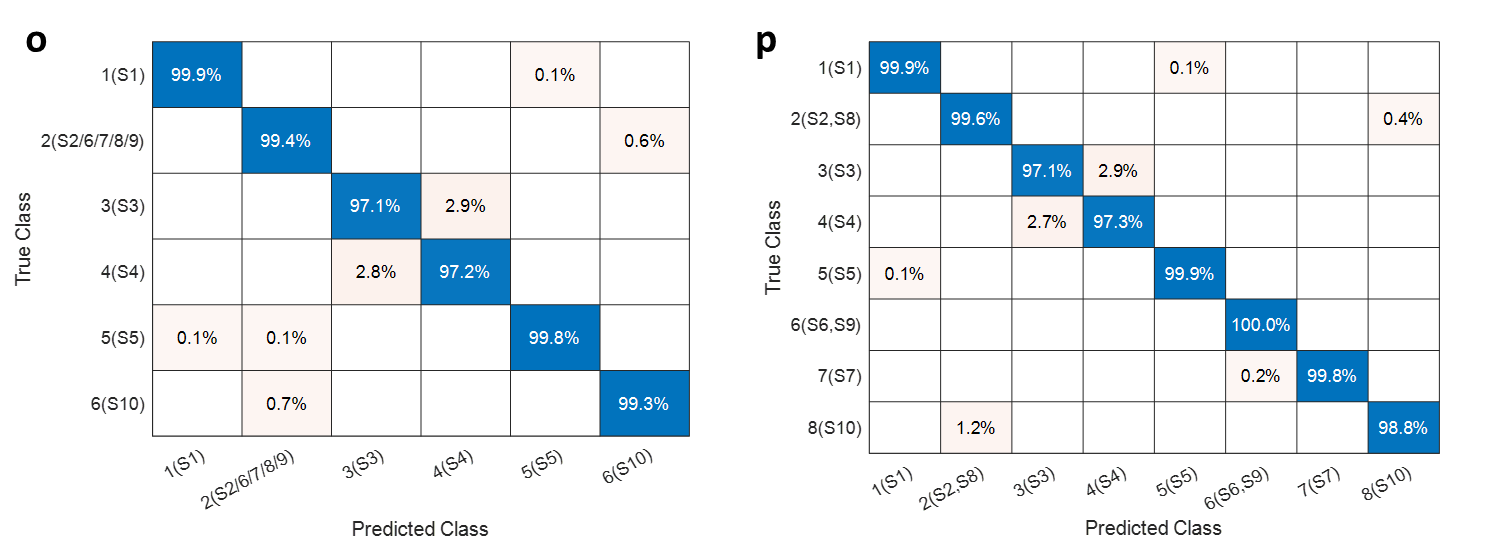

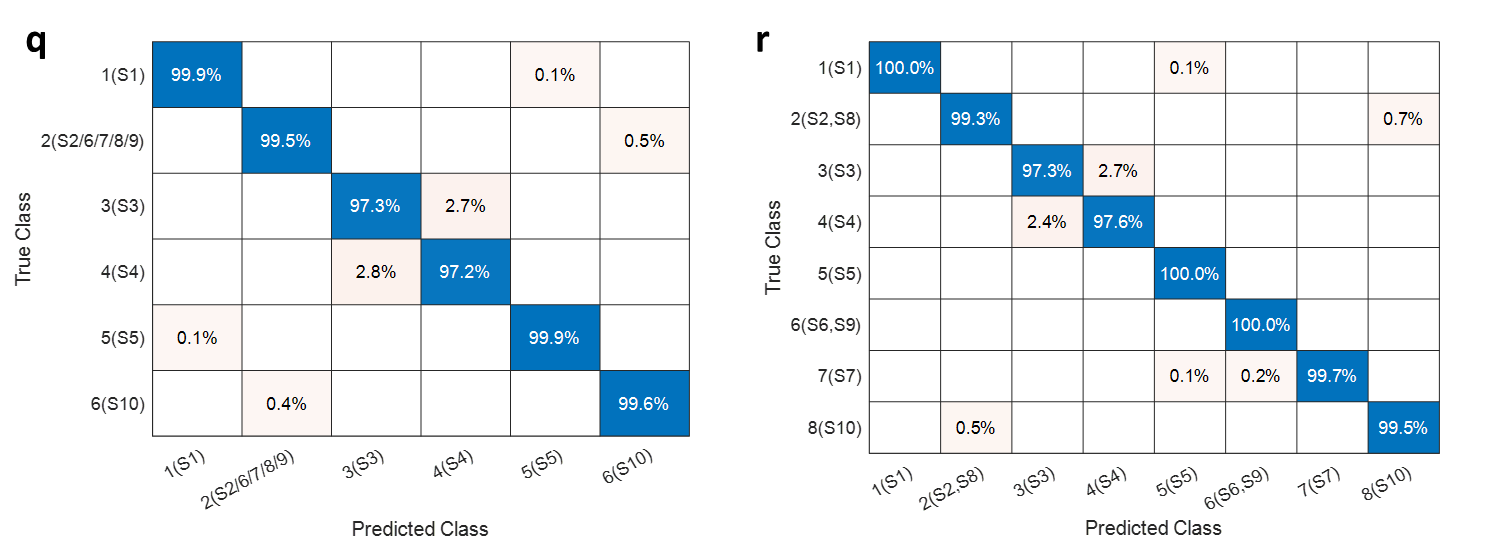

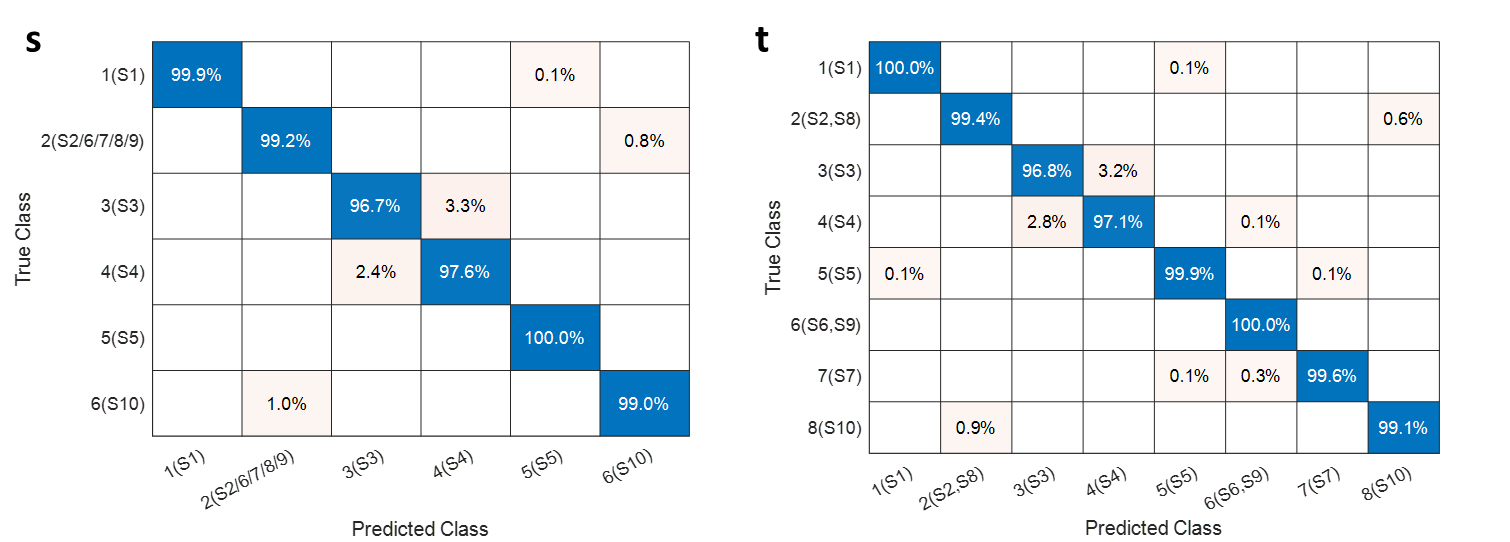

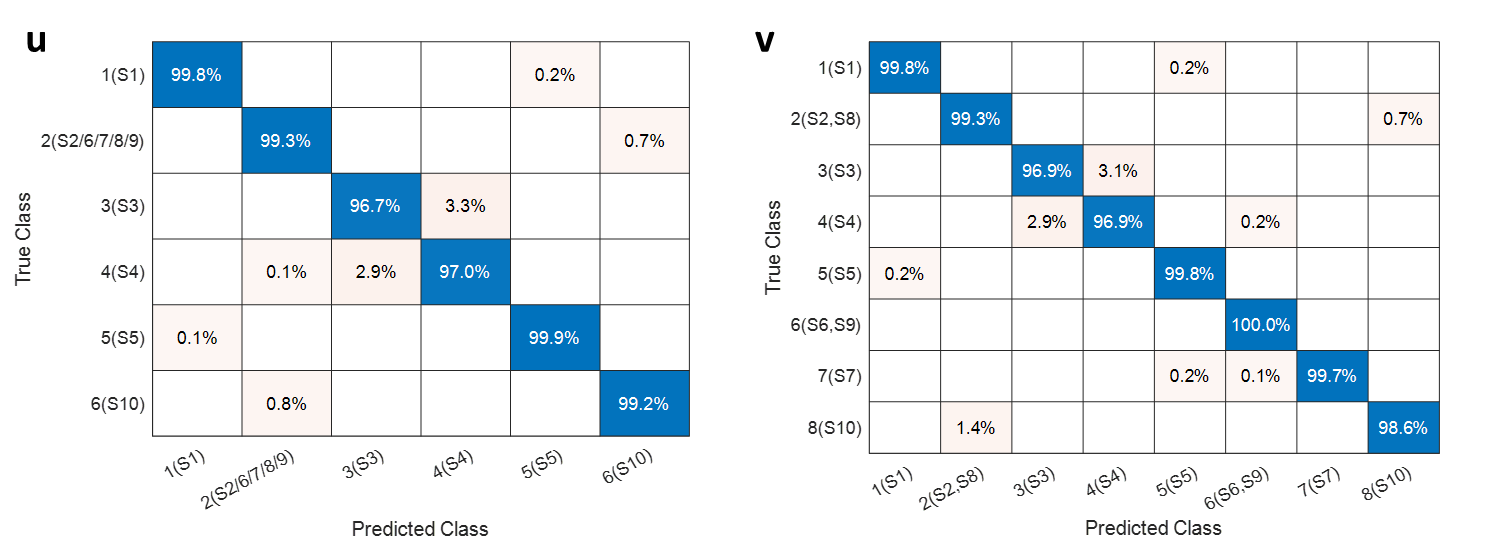

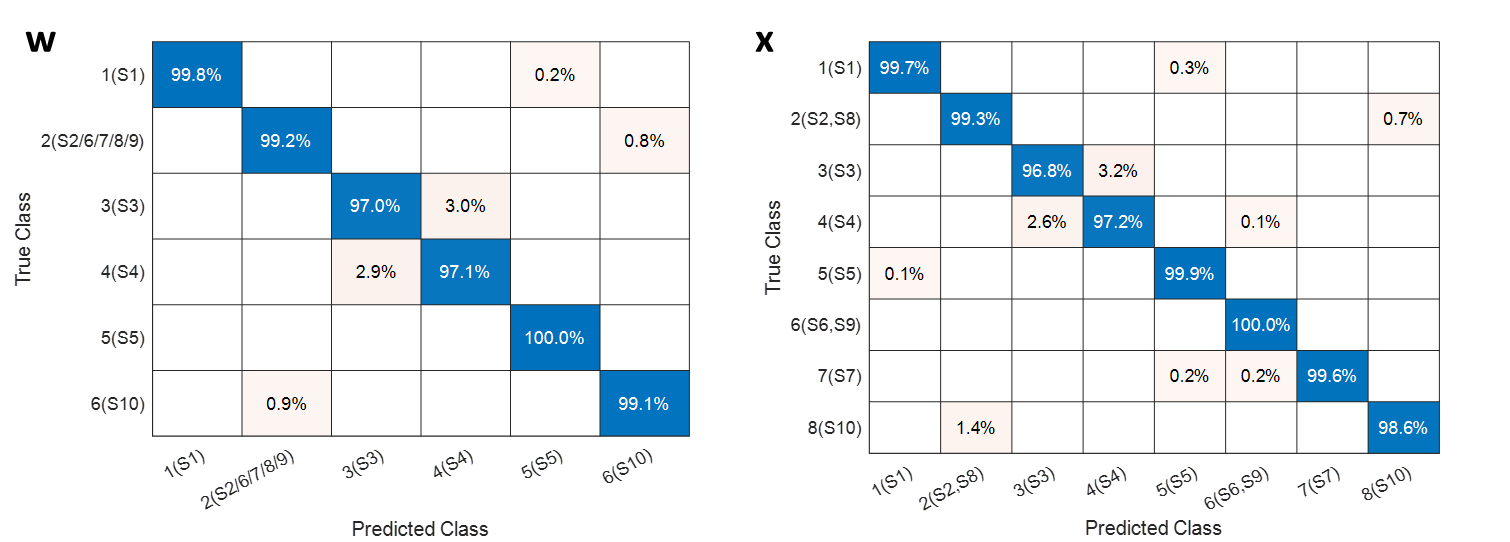

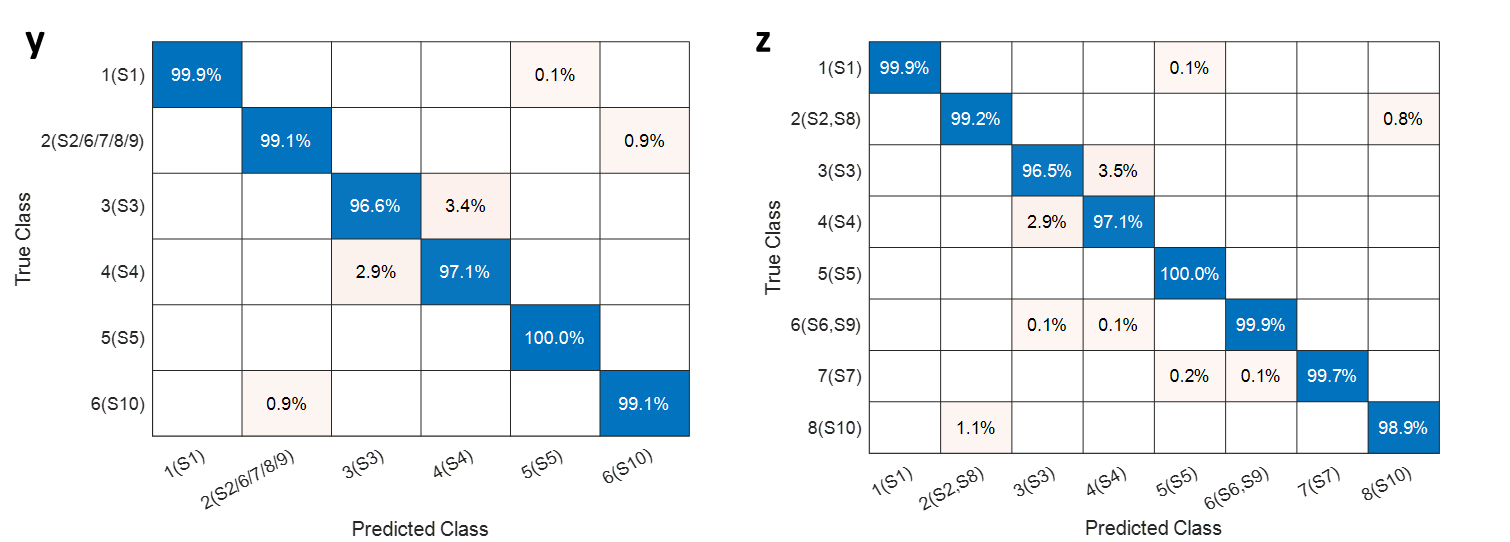

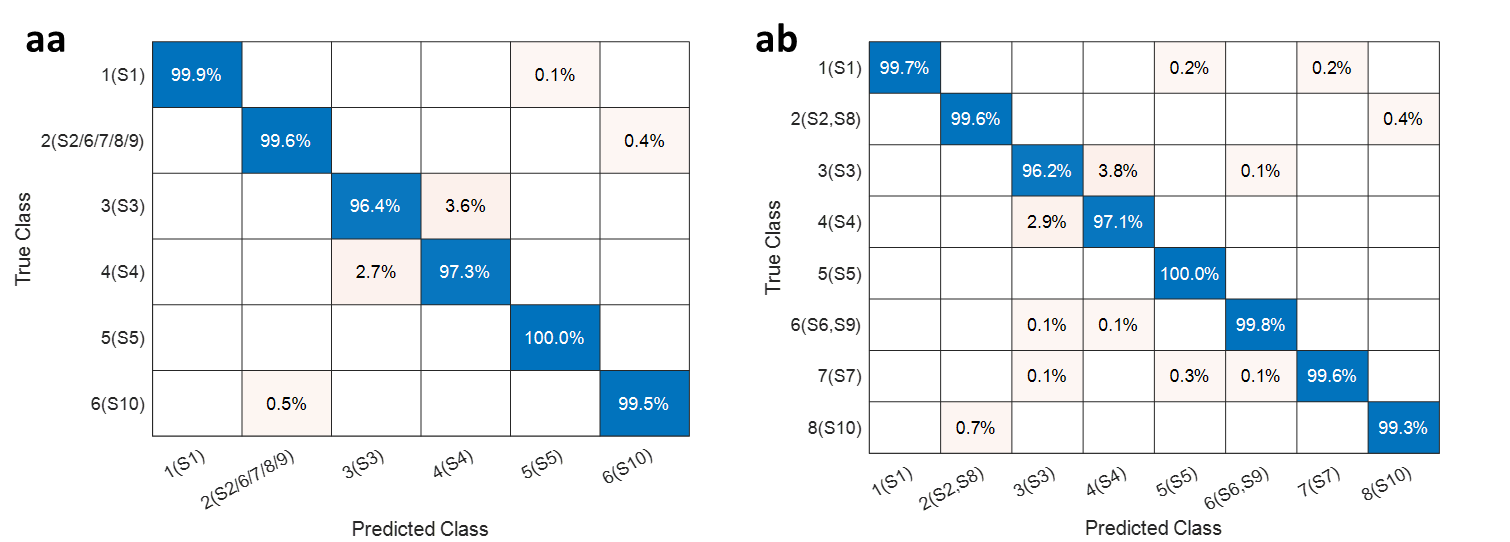

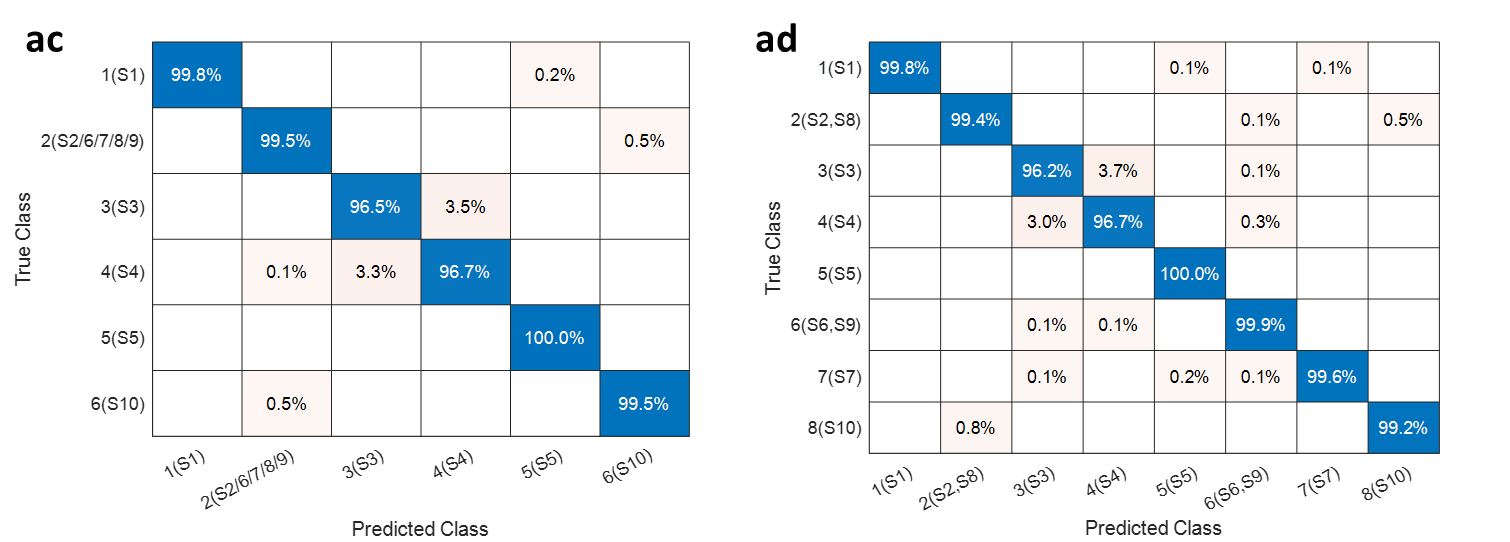


**Figure S9. Performance analysis of baseline classifiers with respect to number of histogram bins, *N_bins_*.** The left-column and right-column figures correspond to TLS-1 with 6 classes and TLS-2 with 8 classes respectively. The confusion matrices correspond to baseline parameter values (*β* = 0.95, *H* = 30) and changing parameter *N_bins_*. (a), (b) for *N_bins_* = 10. (c), (d) for *N_bins_* = 20. (e), (f) for *N_bins_* = 30. (g), (h) for *N_bins_* = 40. (i), (j) for *N_bins_* = 50. (k), (l) for *N_bins_* = 60. (m), (n) for *N_bins_* = 75. (o), (p) for *N_bins_* = 100. (q), (r) for *N_bins_* = 120. (s), (t) for *N_bins_* = 150. (u), (v) for *N_bins_* = 200. (w), (x) for *N_bins_* = 300. (y), (z) for *N_bins_* = 400. (aa), (ab) for *N_bins_* = 500. (ac), (ad) for *N_bins_* = 600 (same as Figure 3 in the main article).


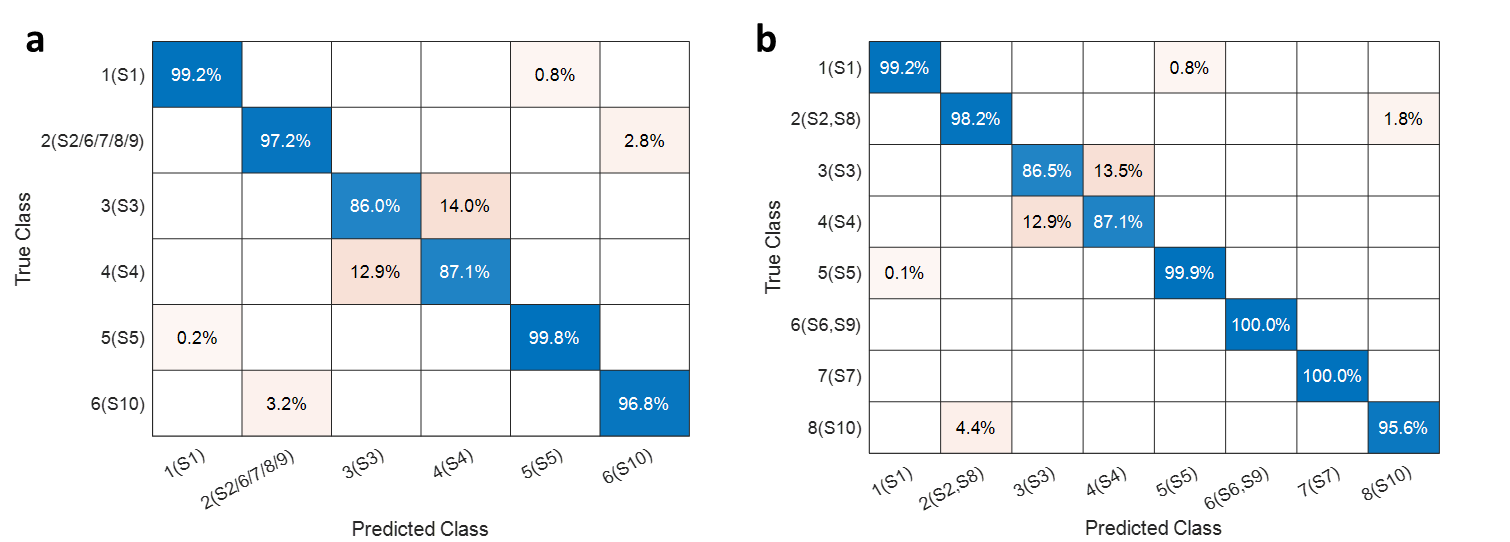

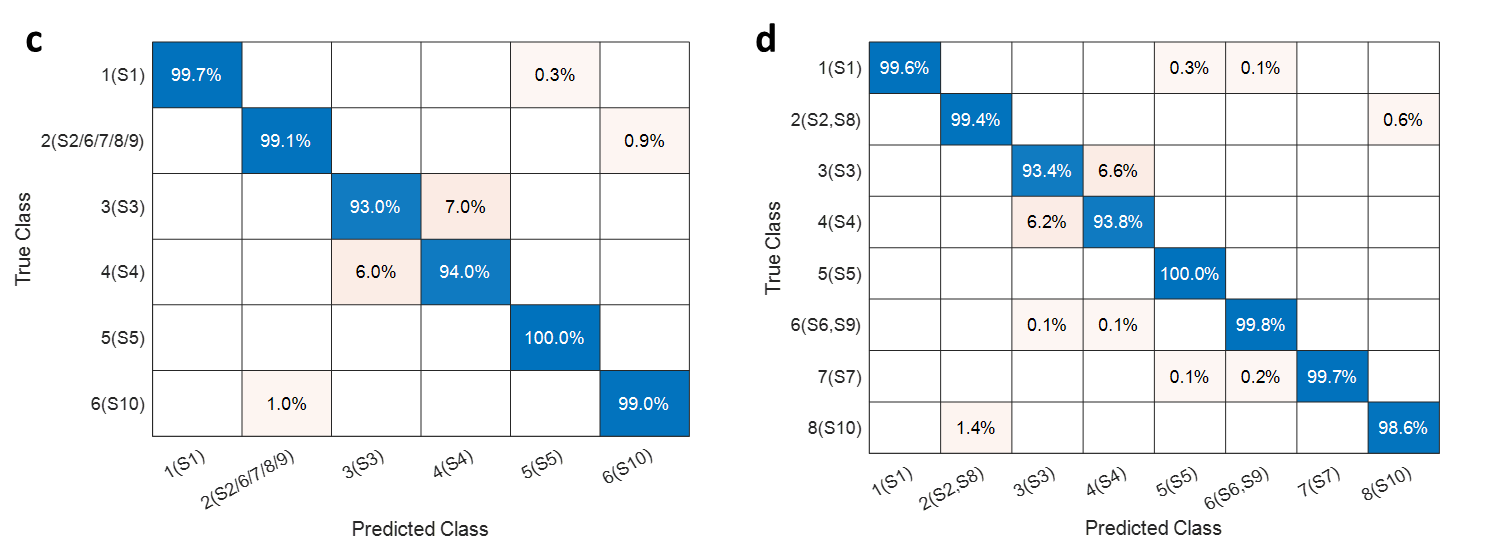

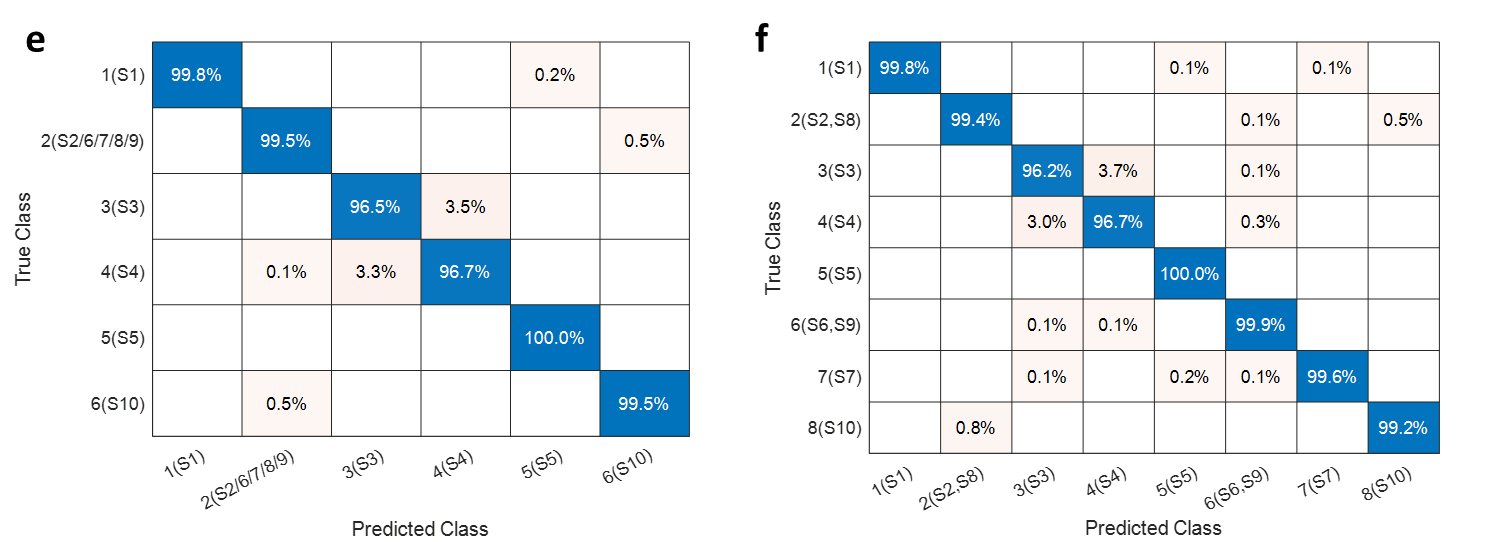

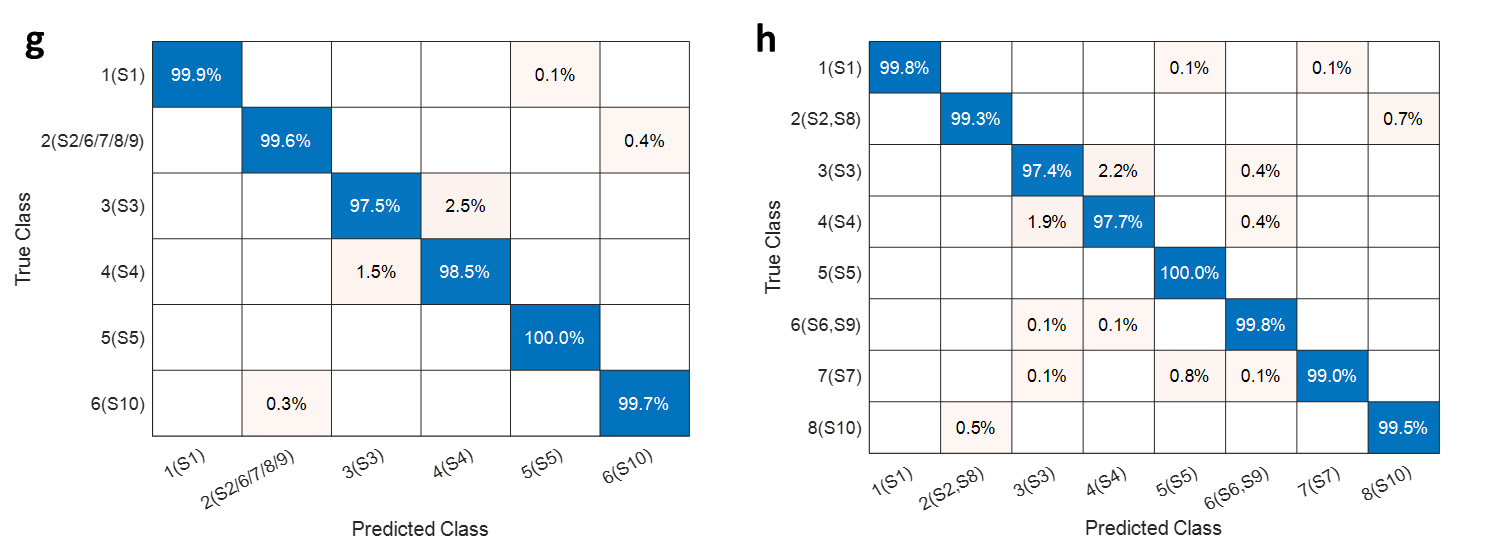

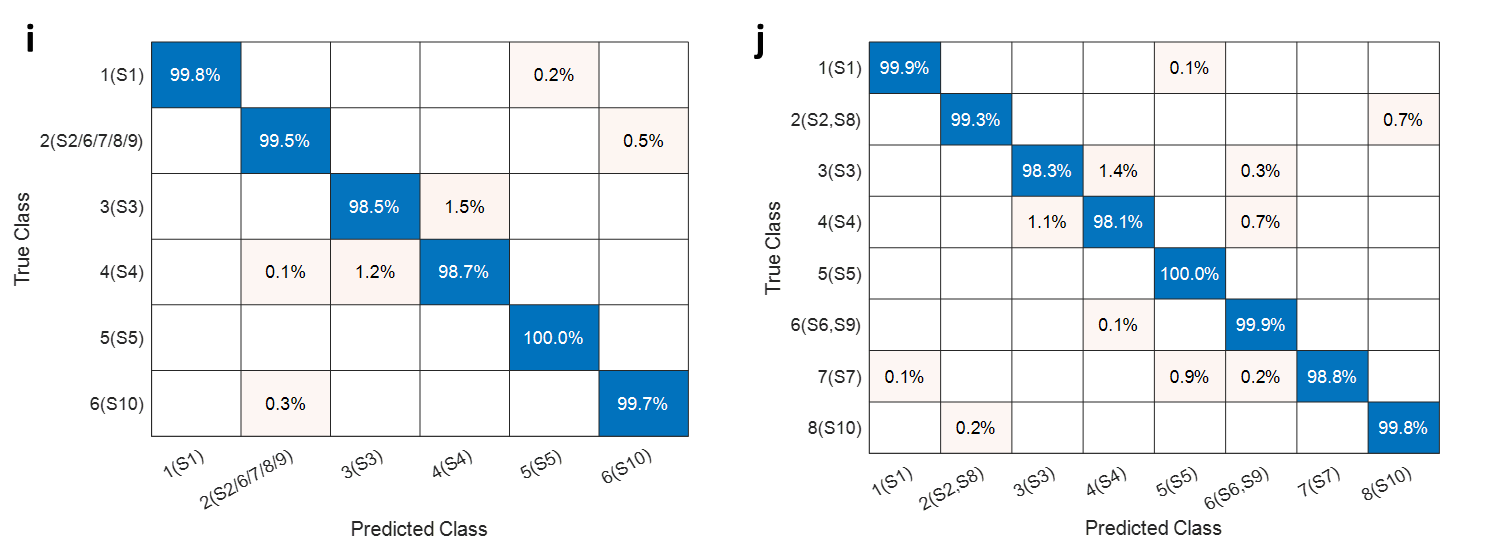


**Figure S10. Performance analysis of baseline classifiers with respect to the number of traces used to compute a conductance histogram, *H.*** The left-column and right-column figures correspond to TLS-1 with 6 classes and TLS-2 with 8 classes respectively. The confusion matrices correspond to baseline parameter values (*β* = 0.95, *N_bins_* = 600) and changing parameter *H*. (a), (b) for *H* = 10. (c), (d) for *H* = 20. (e), (f) for *H* = 30 (same as Figure 3 in the main article). (g), (h) for *H* = 40. (i), (j) for *H* = 50.

**SI References**

[1] Van Der Maaten L, Hinton G. Visualizing Data using t-SNE. vol. 9. 2008.

[2] Wattenberg M, Viégas F, Johnson I. How to Use t-SNE Effectively. Distill 2016;1. doi:10.23915/distill.00002.

[3] Kobak D, Berens P. The art of using t-SNE for single-cell transcriptomics. Nat Commun 2019;10:1–14. doi:10.1038/s41467-019-13056-x.

[4] Hastie T, Friedman J, Tibshirani R. The Elements of Statistical Learning: Data Mining, Inference, and Prediction. New York, NY: Springer New York; 2001. doi:10.1007/978-0-387-21606-5.

[5] Von Luxburg U. A tutorial on spectral clustering. Stat Comput 2007;17:395–416. doi:10.1007/s11222-007-9033-z.

[6] Ng AY, Ng AY, Jordan MI, Weiss Y. On Spectral Clustering: Analysis and an algorithm. Adv Neural Inf Process Syst 2001;14:849--856.

[7] Li Y, Artés JM, Demir B, Gokce S, Mohammad HM, Alangari M, et al. Detection and identification of genetic material via single-molecule conductance. Nat Nanotechnol 2018;13:1167–73. doi:10.1038/s41565-018-0285-x.
